# Pd-Modified Metal Organic Frameworks Synthesized via Mechanochemical Extrusion: Versatile Materials for Suzuki–Miyaura Cross-Coupling and Electrochemical Hydrogen Evolution Reaction

**DOI:** 10.1021/acssuschemeng.6c03049

**Published:** 2026-06-09

**Authors:** Paola Monaco, Oscar Trentin, Daniel Ballesteros-Plata, Giuseppe Misia, Alessandro Silvestri, Enrique Rodríguez-Castellón, Maurizio Selva, Alvise Perosa, Daily Rodríguez-Padrón

**Affiliations:** † Department of Molecular Science and Nanosystems, 19047 Ca’ Foscari University of Venice, Via Torino 155, 30175 Venezia, Mestre, Italy; ‡ Department of Inorganic Chemistry, Facultad de Ciencias, Instituto Interuniversitario de Investigación en Biorrefinerías I3B, Universidad de Málaga, Campus de Teatinos, 29071 Málaga, Spain; § Section of Chemistry for the Technology (ChemTech), Department of Industrial Engineering, University of Padova, Via Marzolo 9, 35131 Padova (PD), Italy

**Keywords:** metal−organic frameworks, mechanochemical extrusion, heterogeneous catalysis, cross-coupling reactions, hydrogen evolution reaction

## Abstract

Extrusion-based mechanochemistry offers a sustainable route for materials synthesis. Here, we report the preparation of Pd-modified metal–organic frameworks (UiO-66, UiO-66-NH_2_, and MOF-801) via continuous-flow twin-screw extrusion. The resulting amorphous Pd@MOF materials contain quasi-spherical Pd nanoparticles whose dispersion is strongly influenced by the functional groups of the parent MOFs, with UiO-66-NH_2_ providing superior stabilization. The catalysts were evaluated in two applications: the Suzuki–Miyaura cross-coupling reaction and the electrochemical hydrogen evolution reaction (HER). Optimization of the Suzuki–Miyaura conditions afforded improved temperature and time parameters compared to literature examples, delivering higher TOFs and meeting CHEM21 First Pass sustainability criteria. A substrate scope analysis, including aryl bromides and chlorides, further confirmed their efficiency, while recycling studies demonstrated catalyst stability. In HER, the highly dispersed Pd nanoparticles maximized active-site availability, with 5Pd@UiO-66-NH_2_-2–200 showing the best performance. The presence of amino groups in the UiO-66-NH_2_ support provided an electron-rich environment that enhanced the intrinsic kinetic rate (*j*_0_). Overall, extrusion mechanochemistry enables robust, versatile Pd@MOF catalysts for both organic synthesis and energy-related applications.

## Graphical abstract

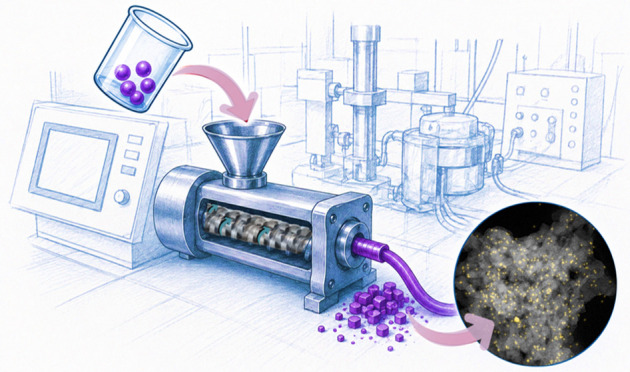

## Introduction

Only a few months before the submission of this work, Kitagawa, Robson, and Yaghi were honored with the 2025 Nobel Prize in Chemistry for the development and systematic investigation of metal–organic frameworks (MOFs); a timely recognition for a field that continues to shape modern materials chemistry.^1–3^ MOFs can be defined as hybrid organic–inorganic two or three-dimensional coordination networks,^4–6^ featuring accessible cavities and a porous structure that can be tuned by the choice of the organic ligands and the metal nodes.^7–9^ Such unique control over morphological, textural, and chemical properties enables the intelligent design of materials for a wide range of applications, including catalysis,^10^ gas adsorption,^11,12^ drug delivery,^13^ biosensing and bioimaging,^14,15^ as well as electrochemical applications and energy storage.^16,17^

Typically, MOFs have been described as highly crystalline materials, although, according to the IUPAC definition, crystallinity is not a key requirement for their classification. Indeed, research on noncrystalline MOFs has recently awakened, driven by their promising possibilities in several fields, particularly in catalysis. In this sense, the catalytic performance of amorphous materials has often resulted in superior performance, in comparison with their crystalline counterparts, most likely due to their higher flexibility, higher number of defects, and exposed active sites. Amorphous MOFs combine the advantages of crystalline MOFs, such as high surface area and tunable porous architectures, with the features of amorphous materials, including isotropy, high density of defects, and active sites.^18–20^

Over the last 25 years, MOFs have been a widely investigated topic within the academic community; still, their translation into scalable applications and commercial products remains a challenge.^21^ Certainly, scaling-up requires careful consideration of the synthetic protocol, along with the associated cost, environmental footprint, and material properties. Several methods have been reported to date for MOFs synthesis, including the traditional solvothermal synthesis, as well as microwave-assisted, electrochemical, and mechanochemical approaches.^22–28^

Among the aforementioned methods, mechanochemical synthesis stands out by its sustainability, allowing a drastic reduction or even the elimination of solvents. Mechanochemistry, although used since ancient times, has been rediscovered in recent years, among other efforts, to move toward a greener chemistry. Mechanochemical synthesis has proven to be effective not only in the formation of nanostructured materials^29–31^ but also in enabling amorphization and defects creation, which ultimately favors catalytic applications.^32,33^ Among mechanochemical methods, reactive extrusion was recognized in 2019 by the IUPAC as one of the top ten technologies poised to change chemistry. Indeed, reactive extrusion enables operation under continuous-flow regimes, facilitating the scalability of the process.^34^ In this sense, the pioneering work by Crawford and James laid the foundation for the extrusion synthesis of MOFs, including HKUST-1, ZIF-8, and Al­(fumarate)­(OH).^35^ Reactive extrusion was later extended to other framework materials, such as UiO-66, UiO-66-NH_2_, MOF-801, and MOF-804.^36^ Compared to conventional solvothermal MOF synthesis, reactive extrusion minimizes solvent consumption, shortens reaction times, and eliminates the need for high-pressure reactors. Furthermore, unlike batch mechanochemical approaches such as ball milling, extrusion operates under continuous-flow conditions, allowing improved control over mixing, residence time, and process reproducibility, while facilitating scale-up and industrial implementation.

Our research group has recently developed extrusion protocols for the synthesis of supported palladium nanoparticles on biomass-derived nitrogen-doped carbonaceous supports, achieving elevated surface areas, close to 500 m^2^/g, and enabling the formation of well-dispersed nanoparticles.^37^ Building on our previous work and on the principles of MOF synthesis via extrusion, this work introduces the extrusion-assisted preparation of Pd-modified MOF-based materials, namely, based on UiO-66, UiO-66-NH_2_, and MOF-801. Unlike conventional solvent-based or postsynthetic routes, the direct incorporation of active metal species within the extrusion process promotes the formation of defect-enriched, catalytically active materials under continuous and solvent-free conditions, thus unlocking new possibilities for scalable, green synthesis of functional hybrid catalysts.

Two different synthetic approaches were explored: a one-step and a two-step synthesis of palladium-modified MOFs, which were labeled as 5Pd@MOF-1–200 and 5Pd@MOF-2–200, respectively (Figure 1). In addition, the catalytic activities of thermally treated samples, 5Pd@MOF-1–500 and 5Pd@MOF-2–500, were also evaluated in this work. Indeed, previous reports on MOF-mediated syntheses^38^ highlighted that the use of higher temperatures enables the formation of carbon-based materials^39^ with well-defined morphologies and highly dispersed metallic nanoparticles, suitable for catalytic and electrocatalytic applications,^40,41^ as well as for supercapacitors and Li-ion batteries.^42^

**Figure 1:**
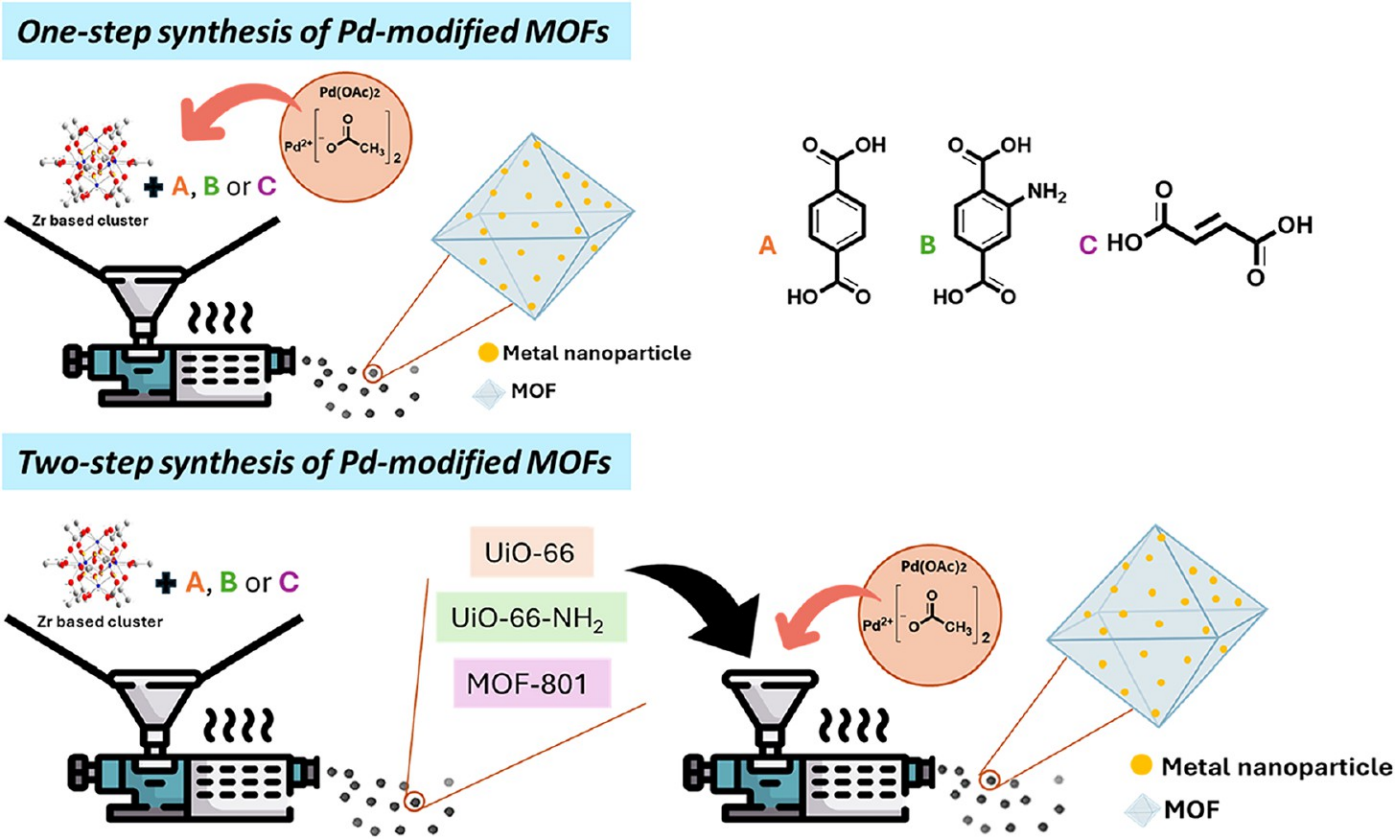
Schematic representation of the designed one-step and two-step synthesis of palladium-modified MOFs.

The catalytic materials designed in this contribution were evaluated in two relevant applications: (i) the Suzuki–Miyaura cross-coupling reaction and (ii) the electrocatalytic hydrogen evolution reaction (HER), demonstrating notable versatility. On the one hand, cross-coupling reactions, recognized with the 2010 Nobel Prize in Chemistry awarded to Heck, Ei-ichi Negishi, and Suzuki, have become highly relevant for the synthesis of complex organic molecules, especially for the pharmaceutical industry.^37,43–45^ On the other hand, HER holds a significant importance as a half-reaction in water splitting, giving access to green hydrogen toward renewable energy technologies.^46,47^

## Results and Discussion

### Materials Characterization

The preparation of palladium-functionalized MOF materials was carried out using a sustainable mechanochemical approach via reactive extrusion, focusing on three zirconium-based architectures: UiO-66, UiO-66-NH_2_, and MOF-801. Unlike traditional solvent-based methods, this continuous-flow protocol allowed for the synthesis of the hybrid catalysts under solvent-free conditions. As previously mentioned, two distinct synthetic strategies were investigated to incorporate the active metal phase: a one-step approach and a two-step approach. In the former, the metal precursor was processed simultaneously with the MOF building blocks, while in the latter, palladium was incorporated into a preformed MOF matrix. In both cases, ethylene glycol was employed as a mild reducing agent to promote the *in situ* formation of Pd nanoparticles during the extrusion process.

A complete characterization of the extruded MOF-based catalytic samples was carried out by using X-ray diffraction (XRD), X-ray photoelectron spectroscopy (XPS), N_2_ physisorption, and high-resolution transmission electron microscopy (HR-TEM). This comprehensive analysis aimed to correlate the structural, textural, and chemical properties of the samples with their catalytic performance in both the Suzuki–Miyaura coupling and electrochemical HER.

The crystal structure and arrangement of the extruded materials were evaluated by XRD analysis (Figure 2). All three prepared MOFs exhibited two main characteristic diffraction peaks at 7.5° ± 1.0 and 8.5° ± 1.0, associated with the (111) and (200) planes of zirconium-based MOFs (ref code: 96–451–2073), corroborating the formation of the desired crystal structures via extrusion.^48–55^ In the case of 5Pd@MOF-1–200 samples, clear differences were observed, especially for the 5Pd@UiO-66–1–200 material, with the presence of sharper peaks. Moreover, the anomalous inversion of the relative intensities of the (200) and (111) reflections, in the 5Pd@UiO-66–1–200 sample, could be related to the concomitant presence of the palladium precursor during the MOFs synthesis. Pd species could most likely modify the MOFs formation kinetics, promote the formation of defects, induce orientation effects, or change the electron density of the Zr clusters, all leading to a decrease of the (111) signal.^56^

**Figure 2:**
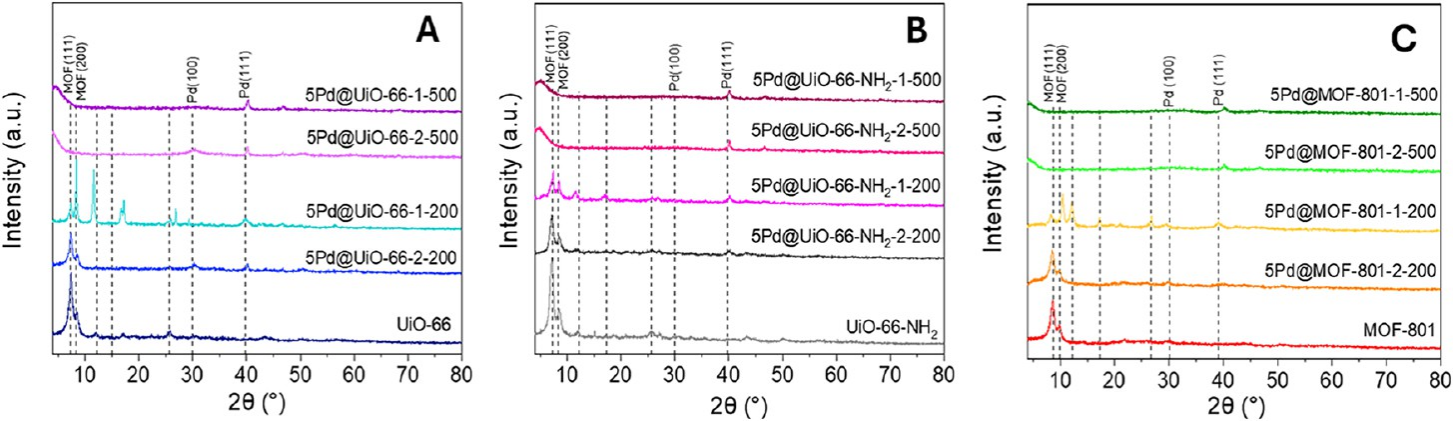
XRD patterns of the mechanochemically prepared (A): UiO-66, (B): UiO-66-NH_2_, and (C): MOF-801-based materials.

Additional broad peaks were also observed around 12.0°, 15.0°, and 25.0°, attributed to the (220), (400), and (442) crystallographic planes of zirconium-based MOFs, respectively.^57^ The low intensity of the previously mentioned peaks confirmed the amorphous nature of the synthesized samples. Furthermore, XRD patterns of the 5Pd@MOF-1–200 and 5Pd@MOF-2–200 samples displayed additional, low-intensity broad peaks around 30.0° and at 39.5°, the second being more evident. These peaks correspond respectively to the (100) plane of PdO and to the (111) plane of Pd(0) with a face-centered cubic crystal structure.^58^ In this case, the low intensity of the palladium-related signals could be indicative of the formation of low particle size nanoparticles or clusters.

Finally, in the case of the 5Pd@MOF-1–500 and 5Pd@MOF-2–500, the disappearance of the reflections related to the (111) and (200) planes of zirconium-based MOFs, together with the appearance of a new pronounced signal at low angle (2θ ∼ 5°), indicated the collapse of the MOF architecture. Such observations were consistent with the retention of mesoscopic order or with a partially preserved framework superlattice.

Furthermore, the textural properties of the materials were evaluated by N_2_ physisorption analysis at 77 K (see Figure 3). The samples mainly displayed Type IV isotherms.^59,60^ In the case of the UiO-66-based samples, the bare MOF displayed a Type IV isotherm with a Type II hysteresis loop, associated with the formation of a mesoporous architecture (Figure 3A). On the other hand, upon palladium incorporation, the hysteresis loop evolved toward a Type III profile, in particular for the 5Pd@UiO-66–1–200 material (Figure 3B).^61,62^ These results suggest a variation in the pore connectivity and a partial occupation of the mesoporous network by Pd entities. In addition, the 5Pd@UiO-66–2–500 displayed a similar isotherm profile to that of the parent 5Pd@UiO-66–2–200 material, with a clear decrease in the adsorbed/desorbed N_2_ volume. Indeed, surface area, pore volume, and pore diameter values also reflect this behavior (Table S1), passing from 498 m^2^g^–1^ in the UiO-66, to 319, 214, and 76 m^2^g^–1^ for 5Pd@UiO-66–2–200, 5Pd@UiO-66–1–200, and 5Pd@UiO-66–2–500, respectively. Moreover, the pore volume was 0.3 cm^3^g^–1^ for the UiO-66, 5Pd@UiO-66–2–200, and 5Pd@UiO-66–1–200 samples, while it decreased down to 0.1 cm^3^g^–1^ for the 5Pd@UiO-66–2–500 material, due to the breakdown of the framework architecture upon thermal treatment at 500 °C. In addition, the mean pore diameter increased with both palladium incorporation and thermal treatment, indicating the partial occlusion or collapse of the smaller pores.

**Figure 3:**
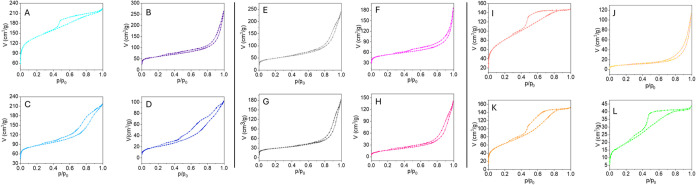
N_2_ physisorption isotherms of the prepared materials. Bare UiO-66 (A); 5Pd@UiO-66–1–200 (B), 5Pd@UiO-66–2–200 (C), 5Pd@UiO-66–2–500 (D), Bare UiO-66-NH_2_ (E); 5Pd@UiO-66-NH_2_-1–200 (F), 5Pd@UiO-66-NH_2_-2–200 (G), 5Pd@UiO-66-NH_2_-2–500 (H), MOF-801 (I), 5Pd@MOF-801–1–200 (J), 5Pd@MOF-801–2–200 (K), and 5Pd@MOF-801–2–500 (L).

Moreover, all UiO-66-NH_2_-based materials exhibited type IV isotherms with a type III hysteresis loop. Textural properties displayed a similar trend, with a decrease in surface area and pore volume and an increase in mean pore diameter, with the exception of the one-pot prepared sample 5Pd@UiO-66-NH_2_-1–200, which showed a higher surface area and lower mean pore diameter (Table S1). Such an anomalous behavior could be attributed to the combination of improved crystallinity, according to XRD results, and possible interactions between Pd precursors and the amine groups of aminoterephthalic acid during synthesis, which could influence the nucleation and growth of the MOF.

Finally, MOF-801-based displayed a similar trend, with Type IV isotherms and Type II hysteresis loop, except for the one-pot prepared material, which displayed a Type III hysteresis. In addition, textural properties, reported in Table S1, displayed a decrease in surface area and pore volume and an increase in mean pore diameter, consistent with the incorporation of palladium nanoparticles and the performed thermal treatment.

HR-TEM images of the prepared samples are shown in Figure 4, corroborating their expected amorphous nature. Particularly, in the case of MOF-801, a certain porosity was evidenced. For the palladium-functionalized materials, micrographs revealed the formation of well-dispersed, quasi-spherical Pd nanoparticles homogeneously distributed on the MOF matrix. Specifically, in the case of 5Pd@UiO-66–1–200 and Pd@UiO-66-NH_2_-1–200, the presence of overlapped square-like layers was observed, consistent with the ordered morphology of the MOFs and in accordance with XRD data, which suggested that, in particular, the one-step synthesized MOFs exhibited a relatively higher crystallinity.

**Figure 4:**
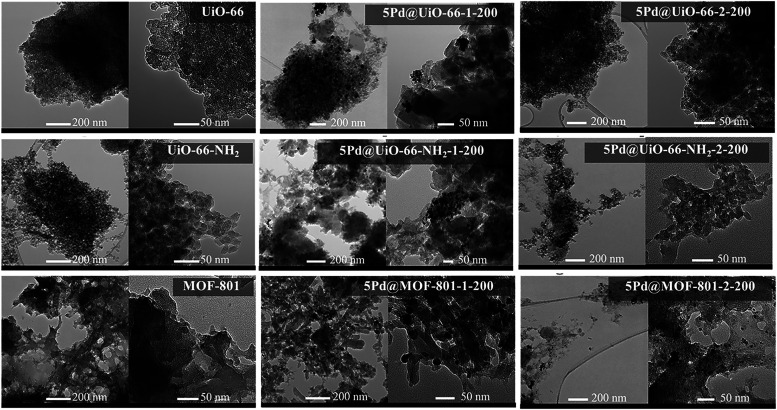
HR-TEM of the MOF-based materials prepared by extrusion.

EDX mapping and STEM micrographs of the parent MOF samples are reported in Figures S1–S3, in the electronic Supporting Information file (SI), confirming the presence of zirconium, carbon, and oxygen; and, in the case of the UiO-66-NH_2_, the additional presence of nitrogen. Moreover, STEM-EDX-mapping analyses of 5Pd@UiO-66–2–200, 5Pd@UiO-66-NH_2_-2–200, and 5Pd@MOF-801–2–200 were performed, as shown in Figure 5. Both 5Pd@UiO-66–2–200 and 5Pd@UiO-66-NH_2_-2–200 samples displayed similar features, with the formation of well-dispersed palladium nanoparticles of approximately 3.8 and 4.0 nm, respectively, and a narrow particle size distribution. Notably, the Pd particle size distribution for Pd@UiO-66-NH_2_-2–200 presents a dominant population of small nanoparticles (1.0–2.0 nm) together with a contribution of larger Pd particles (5.0–6.0 nm), resulting in an asymmetric distribution. In contrast, slightly larger Pd nanoparticles of around 4.9 nm, with a relatively wider particle size distribution, were observed for the 5Pd@MOF-801–2–200 material. The latter material also exhibited certain agglomerated regions, most likely due to the weaker Pd-support interactions in the fumarate-based MOF, as compared to the terephthalate-based analogues. In MOF-801, the absence of functional groups capable of coordinating palladium, such as aromatic π systems or amino groups, limits the metal anchoring, rendering Pd entities prone to migration and agglomeration. Additional HR-TEM, EDX mapping, and STEM micrographs are reported in Figures S4–S9.

**Figure 5:**
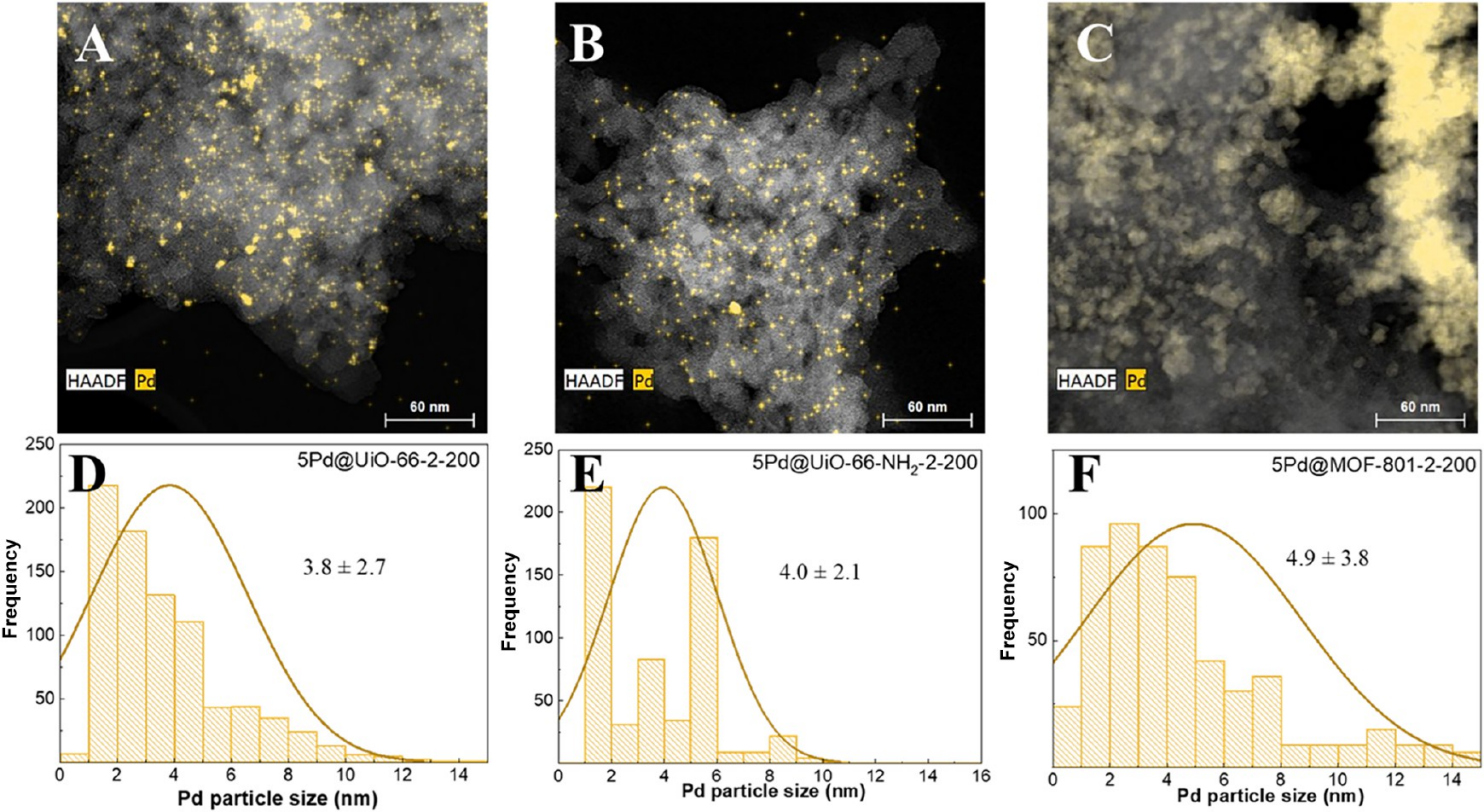
STEM-EDX-mapping micrographs of (A) 5Pd@UiO-66–2–200, (B) 5Pd@UiO-66–NH_2_–2–200, and (C) 5Pd@MOF-801–2–200. The corresponding histograms are shown in (D–F).

The chemical nature of the samples was investigated by XPS analysis, as shown in Figures S10–S12, SI, and in Figure 6. All investigated samples displayed the presence of carbon, oxygen, zirconium, as well as nitrogen in the UiO-66-NH_2_-based materials, and palladium in the Pd-modified counterparts. Deconvolution analysis of the C1*s* core level spectra indicated the presence of three main contributions at ca. 284.6, 286.0, and 288.8 eV, attributed to the presence of C–C/C=C bonds, C–OH/C–O and C=O groups, respectively (Figures S10–S12). Moreover, the O1s core level spectra of the samples exhibited mainly three contributions located at ca. 530.2, 531.9, and 533.9 eV. The lower binding energy components at 530.2 and 531.9 eV are associated with specific oxygen-based species at the Zr_6_-oxo node, namely, Zr–O–Zr and Zr–O–C bonds, respectively.^63^ In line with established XPS literature, the highest binding energy peak at 533.9 eV can likely be assigned to adsorbed moisture trapped within the highly porous MOF architecture.^64^ Moreover, Zr3d core level spectra of all samples were acquired, with Zr3d_3/2_ signal located at ca. 182.9 eV, related to the presence of Zr (IV) species.^65^ In addition, for UiO-66-NH_2_-based materials, N1s XPS spectra showed a signal around 399.5 eV, attributed to the presence of amino groups in the aminoterephthalic acid moieties. In turn, for the 5Pd@UiO-66-NH_2_-2–500 sample, the N1*s* XPS spectrum displayed considerable variations, with the presence of two main contributions, around 398.8 and 400.5 eV, in accordance with the formation of pyridinic and pyrrolic groups, respectively, upon thermal treatment.^66^

**Figure 6:**
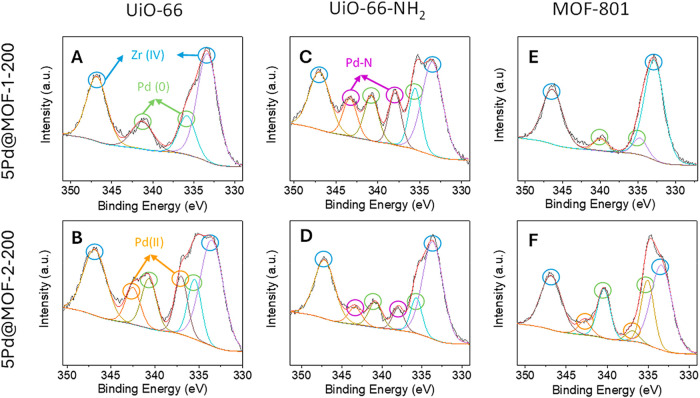
Pd 3d*-*Zr 3p XPS deconvoluted spectra of (A) 5Pd@UiO-66–1–200, (B) 5Pd@UiO-66–2–200, (C) 5Pd@UiO-66-NH_2_-1–200, (D) 5Pd@UiO-66-NH_2_-2–200, (E) 5Pd@MOF-801–1–200, and (F) 5Pd@MOF-801–2–200.

Furthermore, palladium incorporation was corroborated by studying the Pd 3d XPS region (Figure 6). Deconvolution analysis of this region is particularly challenging for this kind of material, due to the overlap with Zr 3p signals. In all cases, contributions at (333.5 ± 0.2) eV and (346.9 ± 0.2) eV were observed, corresponding to Zr 3p_3/2_ and Zr 3p_1/2_ components of Zr (IV), respectively.

In addition, contributions located at (335.5 ± 0.5) eV and (340.5 ± 0.5) eV were identified in all samples, and attributed to the Pd 3d_5/2_-Pd 3d_3/2_ doublet of metallic Pd. Such results confirmed the successful extrusion-assisted *in situ* reduction of palladium species, promoted by ethylene glycol acting as a mild reducing agent. Palladium reduction appeared to be more efficient under the one-step synthetic approach. This can be related to the simultaneous presence of Pd and MOF precursors, along with the reducing agent during extrusion, in contrast with the two-step protocol, where Pd is incorporated into a formed MOF structure and hence mechanical energy is applied to a more rigid architecture, resulting in less complete surface reduction. Indeed, in the case of the UiO-66 and MOF-801 derived samples, prepared by the two-step protocol, the presence of additional signals at (336.5 ± 0.5) eV and (342.3 ± 0.5) eV indicated the coexistence of palladium­(II) species, likely corresponding to residual PdO or Pd^2+^ coordinated to the MOF nodes. Interestingly, in the case of the UiO-66-NH_2_-based samples, either synthesized by the one-step or the two-step protocol, besides the presence of metallic Pd, contributions at ca. (337.5 ± 0.5) eV and (343.3 ± 0.5) eV were observed. These features are consistent with the presence of Pd–N bonds, suggesting coordination of Pd atoms or clusters with the amine functionalities of the organic linker.^37,67^

Quantitative deconvolution of the Pd 3d region for the 5Pd@UiO-66-NH_2_-2–200 catalyst was carried out. After the overlapping Zr 3p contributions were excluded, the relative distribution of Pd species was estimated to be approximately 54% Pd(0) and 46% oxidized/coordinated Pd species.

In summary, the combined characterization techniques reveal that the extrusion-based mechanochemical synthesis promotes the formation of highly dispersed, quasi-spherical Pd nanoparticles (predominantly in the active Pd(0) metallic state) stabilized on the defective amorphous MOF matrices. The small nanoparticle size (around 4 nm) and high dispersion maximize the exposed active surface area, thereby enabling the high turnover frequencies discussed later in the section regarding the Suzuki–Miyaura cross-coupling. Moreover, the homogeneous distribution of these highly accessible Pd(0) sites, coupled with the electron-rich microenvironment provided by specific functional groups (such as the amine moieties in 5Pd@UiO-66-NH_2_), is directly responsible for the enhanced active-site availability and rapid intrinsic kinetics observed during the electrochemical HER, as discussed in the following sections.

### Catalytic Activity: Suzuki–Miyaura Cross-Coupling Reaction

The catalytic systems were tested in the Suzuki–Miyaura cross-coupling between iodobenzene and phenylboronic acid (Scheme 1). In a sealed vial, the reagents were introduced in the following order: 10 mg of catalyst, 0.5 mmol of potassium carbonate, 0.35 mmol of phenylboronic acid, 0.25 mmol of iodobenzene, and 3 mL of ethanol used as a solvent. The mixture was then sonicated for 1 min in an ultrasonic bath to allow a better dispersion of the catalyst, and then stirred at 80 °C for 4 h. The initial reaction conditions were based on previous work conducted by our research group, where these conditions were optimized to lead to a quantitative conversion of the substrate into the desired products.^37^ Ethanol was chosen in that work as an environmentally friendly solvent, since it could be derived from biomass.^68^ The reaction products were identified through GC-MS, while quantification was performed using GC-FID.

**Scheme 1:**
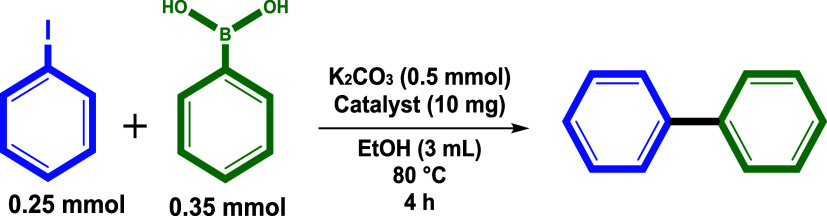
Schematic Representation of the Investigated Suzuki–Miyaura Cross-Coupling Reaction

#### Catalyst Screening

A preliminary catalyst screening was carried out at 80 °C for 4 h to determine which catalytic system was more suitable/gave the best results for the investigated Suzuki reaction. Both the nonpyrolyzed and the pyrolyzed materials were tested, but only the nonpyrolyzed ones gave satisfying results in terms of conversion, selectivity, and yield, measured by GC/FID analysis. As shown in Figure 7, all the tested catalysts exhibited good conversion and yielded quantitative results. To assess the eventual homocoupling of idobenzene, a reaction without the phenylboronic acid was carried out, but it did not lead to any conversion of the substrate.

**Figure 7:**
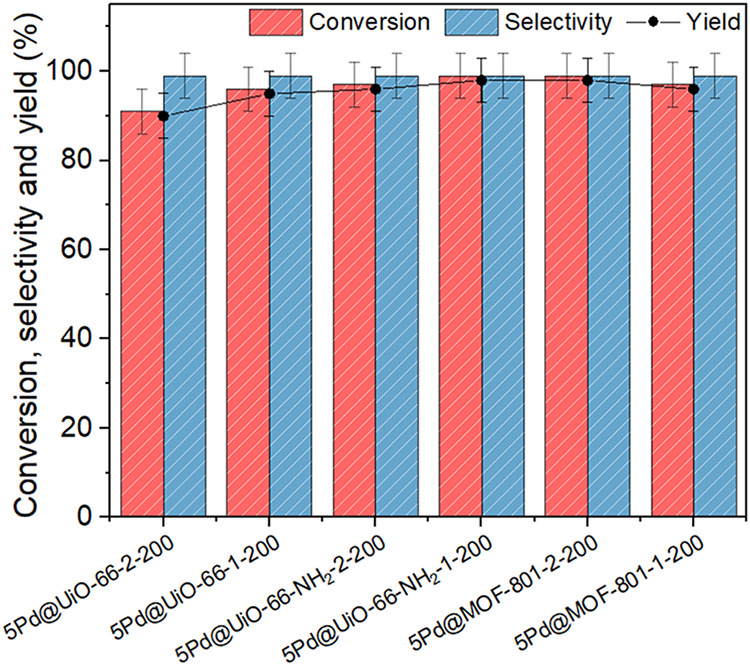
Catalyst screening for the Suzuki cross-coupling reaction. Iodobenzene (1 mmol), phenylboronic acid (0.25 mmol), K_2_CO_3_ (0.5 mmol), catalyst (10 mg), EtOH (3 mL), 4 h, 80 °C.

#### Time Optimization

To verify which is the most active catalyst in the investigated reaction, a study on reaction time was conducted (Figure 8A). All catalytic tests were carried out with the same amount of materials as in Figure 7, but at 60 °C, a temperature deliberately chosen to avoid complete conversion. Operating under these conditions made it possible to discriminate more effectively between the different catalytic materials and to identify the one exhibiting the highest intrinsic activity.

**Figure 8:**
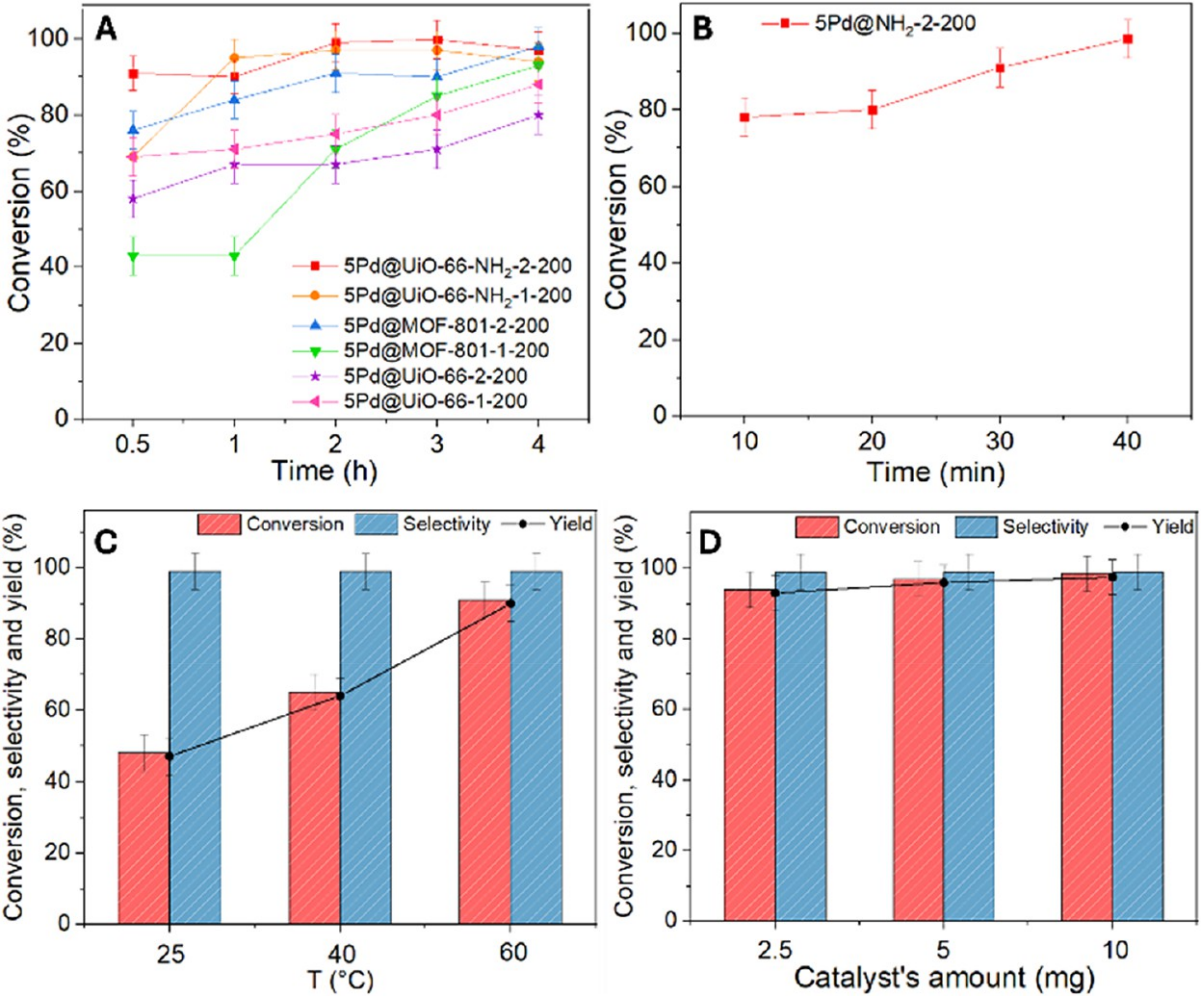
Parametric analysis: Time (A, B), temperature (C), and amount of catalyst (D) optimization study. Iodobenzene (0.25 mmol), phenylboronic acid (0.35 mmol), K_2_CO_3_ (0.5 mmol), EtOH (3 mL). Best condition with 5Pd@UiO-66-NH_2_-2–200, 40 min, 60 °C.

The results indicate that the reaction initiates quite early, and all the materials give more than 40% of conversion after just 30 min. Overall, it was observed that the material exhibiting the best catalytic performance within the shortest reaction time was 5Pd@UiO-66-NH_2_-2–200. This catalyst reached a remarkable 91% conversion after only 30 min, clearly outperforming the other materials under identical reaction conditions. However, after approximately 3 h of reaction, the conversion values obtained for all the investigated MOFs became comparable.

To gain a deeper understanding of the kinetic behavior of the most active material, an additional time-dependent screening was performed (Figure 8B). This experiment aimed to obtain a more detailed reaction profile, extending the analysis to shorter reaction intervals starting from 10 min. The results revealed that 5Pd@UiO-66-NH_2_-2–200 maintains a very high level of activity even over such short time scales, reaching 80% conversion within only 10 min. This outstanding performance highlights its potential as an exceptionally active catalyst for this transformation. Moreover, a quantitative conversion (98.5%) was achieved after only 40 min.

#### Temperature Optimization

With the most active catalytic system, the optimization of the reaction temperature was carried out, as shown in Figure 8C. Experiments were performed under the same condition of Figure 7, except for the time of 30 min, varying the temperature to 25, 40, and 60 °C. The upper temperature limit of 60 °C was selected because preliminary observations had already shown that, at this temperature, an almost complete conversion could be achieved within 30 min. As illustrated in the graph, temperature plays a crucial role in enhancing the reaction yield. Nevertheless, even at room temperature, the substrate conversion reached 45% after only 30 min, confirming the high catalytic activity under mild conditions. As expected, the best performance was obtained at 60 °C, which was therefore chosen for subsequent experiments. This represents an improvement compared with several studies reported in the literature, where an optimal temperature of around 80 °C is typically required to maximize product yield.^69–73^ The optimization of this parameter contributes positively to the overall greenness of the process.^74^

#### Catalyst’s Quantity Optimization

Further optimization focused on reducing the catalyst loading and, consequently, the metal-to-substrate molar ratio. Catalyst amounts of 2.5, 5, and 10 mg were evaluated, corresponding to substrate-to-Pd ratios of 212:1, 106:1, and 53:1, respectively. All experiments were conducted at 60 °C for 40 min, as these conditions had previously afforded the best performance, while maintaining all other parameters identical to those reported in Figure 7. As shown in Figure 8D, even at the lowest catalyst loading (2.5 mg), the reaction delivered an excellent 93% yield of biphenyl. This result is fully consistent with the high intrinsic activity of the MOF-based catalyst, already evident from the time-course optimization. Considering that increasing the catalyst amount affects the conversion by only a few percentage points, well within experimental error, the use of 2.5 mg represents the optimal compromise between catalytic efficiency and productivity, with the reaction performed at 60 °C for 40 min.

### Literature Comparison for Suzuki–Miyaura Cross-Coupling

To assess the catalytic performance of the synthesized material in the Suzuki–Miyaura cross-coupling reaction, a comparative analysis was carried out using representative literature studies. As summarized in Table 1, these systems employ palladium supported on a variety of materials. In addition to the catalyst developed in this work, the comparison includes: (i) a chitin-derived carbon-supported Pd catalyst previously prepared by our group (CNi);^37^ (ii) a Pd complex immobilized on a cellulose-alumina composite obtained via a multistep synthesis;^70^ (iii) Pd nanoparticles supported on a ZnFe_2_O_4_ magnetic core;^73^ (iv) a carbon-bead-based material requiring an energy-intensive thermal treatment at 1500 °C (TOP/MB-1500);^75^ (v) a Pd catalyst supported on a different MOF;^76^ (vi) a system based on polyamide-modified graphene oxide;^77^ and (vii) a nanocomposite obtained through electrochemical impregnation of nanostructured tetragonal ZrO_2_ (Pd-NPs/ZrO_2_).^78^

**Table 1:** Literature Comparison in the Suzuki Cross-Coupling Reaction

entry	catalyst	molar ratio (%)	*T* (°C)	*t* (h)	yield (%)	TOF (h^–1^)	refs
1	5Pd@UiO-66-NH_2_-2–200	0.4	60	0.66	93	297	this work
2	1Pd/CNi	0.32	75	4	97	65	^37^
3	Pd-AMP-Cell@Al_2_O_3_	1.8	80	1	98	54	^70^
4	ZnFe_2_O_4_@SiO_2_@CPTMS@PYA-Pd	0.9	95	1.7	96	66	^73^
5	Pd (TOP)/MB-1500	1	100	4	90	23	^75^
6	MOF-253·0.05PdCl_2_	0.2	100	8	98	53	^76^
7	GO-NH_2_–Pd^2+^	1	60	0.5	87	190	^77^
8	Pd-NPs/zirconia	0.1	90	14	90	64	^78^

Although the reported yields are generally satisfactory across all studies, the corresponding reaction conditions vary substantially. Besides the solvent choice, where DMF and DMSO, used in some cases (Table 1, entries 3, 4, and 6), raise clear sustainability concerns due to their high toxicity, the most relevant parameters for comparison are the Pd-to-substrate molar ratio, reaction temperature, and reaction time. Across these metrics, the catalyst synthesized in the present work operates under more favorable and milder conditions, while also avoiding the energy-intensive preparation steps required by several literature systems. This highlights its potential both as an efficient and as a more sustainable catalytic platform.

To allow a quantitative comparison between catalysts, a turnover frequency (TOF) analysis was performed. The catalytic performance was first assessed by calculating the TOF based on the total Pd content in the material. ICP-OES analysis was performed to verify the actual metal content after synthesis. The experimentally determined Pd contents were found to be slightly lower than the nominal value. Nevertheless, the values remain in good agreement with the targeted loading, being approximately 4.6–4.9 wt %, depending on the catalyst.

Under the optimized reaction conditions (93% yield in 40 min, 2.5 mg catalyst, ca. 5 wt % Pd), this bulk-normalized approximation gives a TOF of 297 h^–1^. While useful for literature benchmarking, this value considerably underestimates the intrinsic activity of the catalyst, as only surface-exposed Pd atoms participate in the Suzuki cycle. A more rigorous estimation was therefore obtained by analyzing the nanoparticle dispersion. TEM images revealed Pd nanoparticles with an average diameter of ca. 4.0 nm, enabling the estimation of the number of superficial Pd atoms per particle and thus of the accessible catalytic sites. Using a spherical particle model and the corresponding size distribution, approximately 17% of Pd atoms lie on the nanoparticle surface, corresponding to 2.0 × 10^–7^ mol of surface Pd under reaction conditions. Normalizing the reaction rate to these accessible sites gives a site-based TOF of approximately 1764 h^–1^. Considering that only a fraction of surface atoms, typically around 10%, associated with edge and defect sites, are catalytically relevant, the intrinsic TOF reaches the 17,640 h^–1^ value. These values are consistent with highly dispersed Pd nanostructures and highlight the remarkable intrinsic activity of the 5Pd@UiO-66-NH_2_-2–200 catalysts, far beyond what is captured by bulk-metal TOF calculations. Further details of the TOF estimation are provided in the SI.

### Sustainability Analysis

The sustainability performance of the 5Pd@UiO-66-NH_2_-2–200 was assessed using the CHEM21 First Pass green metrics toolkit, which evaluates discovery-scale transformations through a combination of mass efficiency, energy input, solvent and reagent hazards, and workup intensity.^74,79^ Within this framework, our protocol meets several green-flag criteria, indicating an intrinsically favorable sustainability profile.

The first positive indicator concerns energy input, a key CHEM21 parameter. Although the toolkit does not impose strict numerical thresholds, reactions performed below 80–100 °C are generally categorized as low-energy, green-flag conditions at First Pass, since they avoid the amber/red classification associated with high-temperature or pressure-intensive operations used in industrial pilot stages. Our reaction proceeds at 60 °C for only 40 min, fitting squarely within the green-flag operating window and substantially reducing heating demand compared to typical Pd-catalyzed Suzuki protocols (80–100 °C).

Regarding chemical hazards, the CHEM21 First Pass explicitly assigns red and amber flags based on GHS safety categories.^80^ The present reaction avoids all substances triggering red-flag classifications (e.g., H300/H310 acute toxicity, H400–H410 long-term aquatic toxicity), and no reagents fall under the “highly explosive” or “explosive thermal runaway” classes (H200–H203, H230). This places the protocol in the green category for chemical hazard burden, a critical early stage requirement to ensure that optimization does not embed inherent risk.

From a mass-efficiency standpoint, the reaction benefits from minimal stoichiometric auxiliaries, and the heterogeneous catalyst eliminates the need for scavengers or extensive aqueous workup. CHEM21 identifies the process mass intensity (PMI) as a foundational quantitative metric. Under our optimized conditions, the reaction exhibits a PMI of 70, which is typical of small-scale solution-phase processes in which the solvent accounts for the majority of the mass input (94%). The high yield (93%) and the absence of significant byproducts result in a favorable reaction mass efficiency (RME = 44%), whereas the atom economy (AE = 47%) reflects the intrinsic stoichiometric efficiency of the Suzuki–Miyaura transformation. Together, these parameters align with the CHEM21 objective of identifying transformations that balance intrinsic reaction efficiency with practical operational performance. These values, along with the favorable conversion, selectivity, and overall efficiency (OE), are summarized in the radar chart highlighting the six key metrics: conversion, yield, selectivity, OE, RME, and AE (Figure 9).

**Figure 9:**
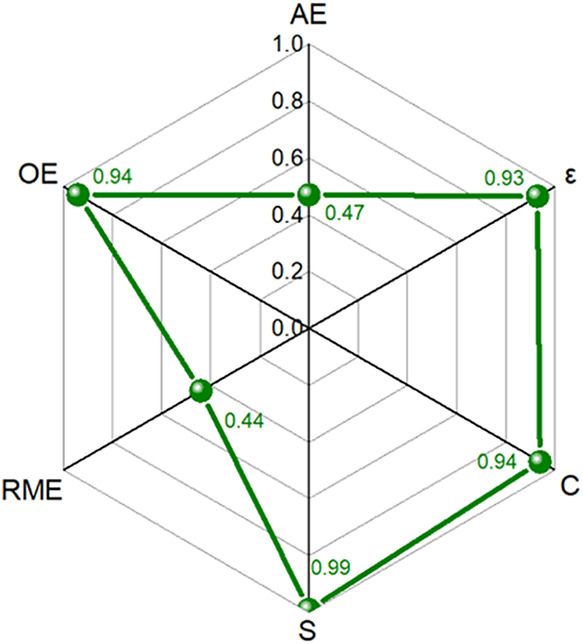
Hexagon radial chart detailing the evaluation of green metrics associated with the performance of the 5Pd@UiO-66-NH_2_-2–200 catalyst in the Suzuki–Miyaura cross-coupling process.

Overall, applying the CHEM21 First Pass criteria shows that the Suzuki coupling with 5Pd@UiO-66-NH_2_-2–200 achieves green flag performance in energy input, hazard profile, and mass efficiency. This establishes a strong sustainability baseline at the discovery scale and identifies the methodology as a promising candidate for subsequent second-pass evaluation during scale-up. Since solvents typically contribute more than half of the overall mass intensity in API synthesis,^81^ reducing solvent usage remains the most effective strategy for enhancing process greenness. In this context, mechanochemistry, particularly reactive extrusion, offers an attractive avenue, combining ultralow solvent demand with inherent scalability and enabling continuous flow processing, which improves mass efficiency relative to that of conventional batch operation.

To preliminarily explore this approach, a mechanochemical experiment was conducted under conditions mirroring the optimal thermal protocol, scaling all reagents 8-fold to provide sufficient material for extrusion. The mixture was processed for 40 min at 60 °C, reaching 35% conversion, substantially lower than under solution conditions. Despite the lower conversion, the experiment confirms that the Pd@MOF catalysts retain measurable activity in a highly solvent-minimized, mechanically driven environment, demonstrating robustness and potential adaptability. Considering the excellent catalytic performance, mild conditions, and favorable CHEM21 First Pass metrics in solution, the methodology already stands on a strong sustainability and efficiency foundation, and future studies aimed at optimizing extrusion or other continuous solvent-minimized processes will build upon an intrinsically high-performing and green catalytic platform.

### Substrate Scope

The substrate scope was evaluated by using various halogenated aryl derivatives and phenylboronic acids (Table 2). As expected, for the aryl derivatives, the reactivity follows the order I > Br > Cl, consistent with the relative C–X bond strengths that decrease the activation energy required for oxidative addition for iodides. Nevertheless, brominated derivatives, which are generally significantly less reactive than iodides, still provided moderate to good yields, highlighting the robustness of the 5Pd@UiO-66-NH_2_ catalyst.

**Table 2:**
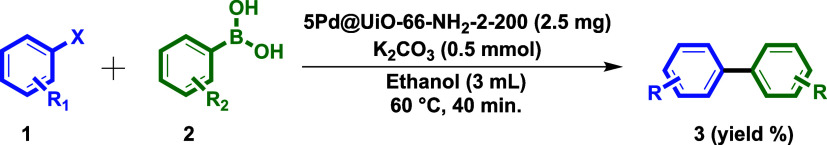

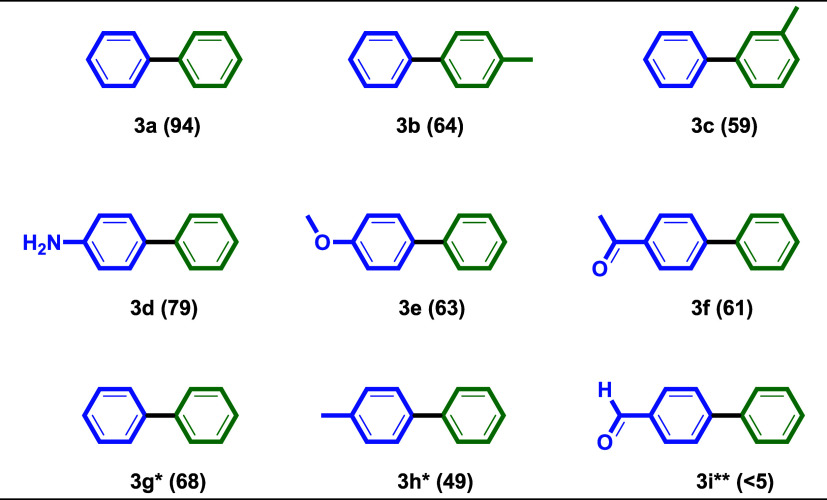
Substrate Scope[Table-fn t2fn3]

* The corresponding aryl bromine was used.

** The corresponding aryl chlorine was used.

a Reaction conditions: Aryl iodide (0.25 mmol), arylboronic acid (0.35 mmol), K_2_CO_3_ (0.5 mmol), 5Pd@UiO-66-NH_2_-2-200 (2.5 mg), ethanol (3 mL), 60 °C, 40 min; yield of product 3 is reported in brackets, calculated by GC/FID.

Interestingly, aryl iodides bearing electron-donating groups (−OMe and −NH_2_) afforded comparable or even higher yields than those containing electron-withdrawing substituents (e.g., −COCH_3_), which contrasts with the trend typically observed in homogeneous Pd catalysis. In homogeneous systems, oxidative addition is generally rate-determining and thus favored by electron-poor substrates. However, for the heterogeneous 5Pd@UiO-66-NH_2_-2–200 catalyst, several factors may reverse this tendency.

First, the Pd nanoparticles are electronically enriched through strong Pd–N interactions with the amino-functionalized UiO-66 support. This increased electron density facilitates oxidative addition even for electron-rich aryl iodides, shifting the rate-determining step toward transmetalation or reductive elimination, both of which are favored by electron-donating groups.

Second, surface adsorption phenomena within the UiO-66-NH_2_ matrix play a major role. The organic linkers of the MOF can engage in π–π stacking interactions with aromatic substrates. Electron-rich aryl halides typically exhibit stronger π–π interactions with the terephthalate linkers, leading to preferential adsorption near catalytically active Pd sites and improved local substrate concentration at the metal–support interface. Conversely, electron-deficient aryl halides interact more weakly, reducing effective surface contact and slowing the overall reaction rate.

Therefore, the combination of electronically promoted Pd centers and π–π stacking-mediated substrate enrichment in the vicinity of active sites provides a consistent rationale for the enhanced reactivity of electron-donating substrates in this heterogeneous system.

The Hammett analysis of the Suzuki–Miyaura coupling catalyzed by 5Pd@UiO-66-NH_2_-2–200 shows a nonlinear correlation, indicating that the reaction does not follow a simple electronic effect trend (Figure S13). When the substrates are divided into electron-donating (−NH_2_, –OCH_3_, −CH_3_) and electron-withdrawing (−COCH_3_, –H) groups, both subsets display negative slopes. This means that within each group, electron-rich aryl halides react faster than less electron-rich or electron-deficient ones, which is the opposite of what is typically observed in homogeneous Pd-catalyzed systems. The negative slopes across both groups suggest that the reaction rate is not solely determined by classical electronic effects on oxidative addition. Instead, the unusual Hammett behavior likely arises from the complex interplay of substrate–catalyst interactions on the heterogeneous MOF surface, such as adsorption and local concentration effects, which can amplify the reactivity of electron-donating aryl halides relative to electron-withdrawing ones. The overall nonlinear plot reflects these combined effects and underscores the distinctive influence of the MOF environment on substrate reactivity.

### Catalyst Recyclability

After identifying the optimal reaction conditions and completing the substrate scope, the stability of the catalytic system was assessed through recycling experiments. The reaction was performed at 60 °C for 20 min using 10 mg of catalyst. A higher catalyst loading was deliberately selected to minimize potential material loss during recovery. At the end of each cycle, the reaction mixture was centrifuged to separate the solid catalyst from the supernatant. The recovered solid was washed twice with diethyl ether, dried, and reused in the subsequent run. To maintain an excess of base relative to the substrate, an equivalent amount of K_2_CO_3_ (0.25 mmol, 35 mg) was added at the beginning of each cycle.

Four additional cycles were carried out after the initial reaction. As shown in Figure 10, the catalyst retained good activity, although a gradual decline in conversion was observed over successive runs: 81, 76, 74, and 70% for the first, second, third, and fourth cycles, respectively. These results demonstrate that the material exhibits good operational stability and preserves its catalytic performance under repeated use. The slight decline in activity may be associated with common deactivation phenomena such as partial palladium leaching, partial blockage of active sites by adsorbed species, or structural alteration of the MOF during repeated cycles.

**Figure 10:**
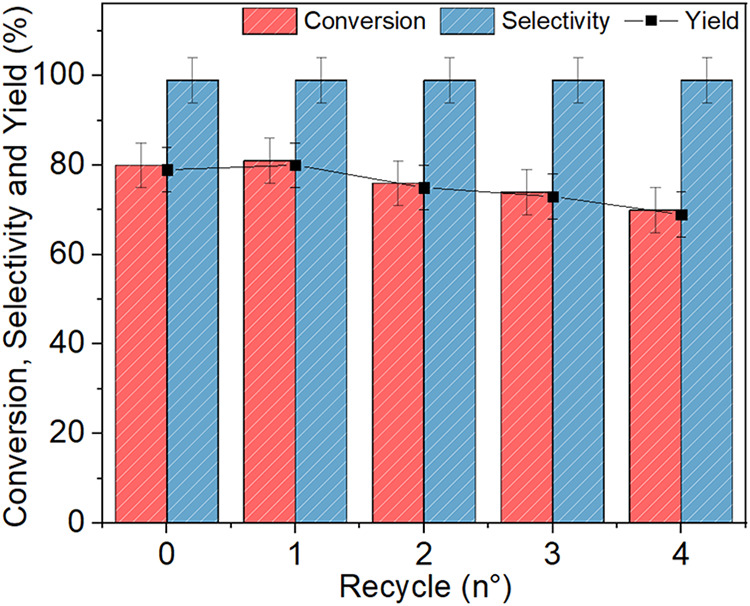
Recyclability study of the Suzuki–Miyaura cross-coupling reaction. Iodobenzene (0.25 mmol), phenylboronic acid (0.35 mmol), K_2_CO_3_ (0.5 mmol), 5Pd@UiO-66-NH_2_-2–200 (10 mg), ethanol (3 mL), 60 °C, 20 min.

To assess the potential contribution of palladium leaching, a Sheldon hot filtration test was performed. A reaction mixture containing iodobenzene (0.25 mmol), phenylboronic acid (0.35 mmol), K_2_CO_3_ (0.5 mmol), and 2.5 mg of catalyst in 3 mL of ethanol was heated at 60 °C for 20 min, reaching a conversion of 67% according to GC analysis. The catalyst was then removed by hot filtration, and the clear filtrate was allowed to react under identical conditions for an additional 20 min. In this second stage, the conversion reached 69%, a difference within the experimental error (±5%). This outcome indicates that no significant homogeneous catalytic contribution is present and supports the predominantly heterogeneous nature of the catalytic system. ICP analysis of the postreaction solution revealed Pd concentrations below the detection limit of the instrument, suggesting that the active phase remains largely immobilized on the support under the reaction conditions. Thus, the moderate decrease in activity is therefore unlikely to arise from Pd leaching. Instead, postreaction HR-TEM/STEM-EDS analyses suggest that catalyst deactivation is more likely associated with a partial Pd nanoparticle restructuring and sintering/agglomeration (Figure 11).

**Figure 11:**
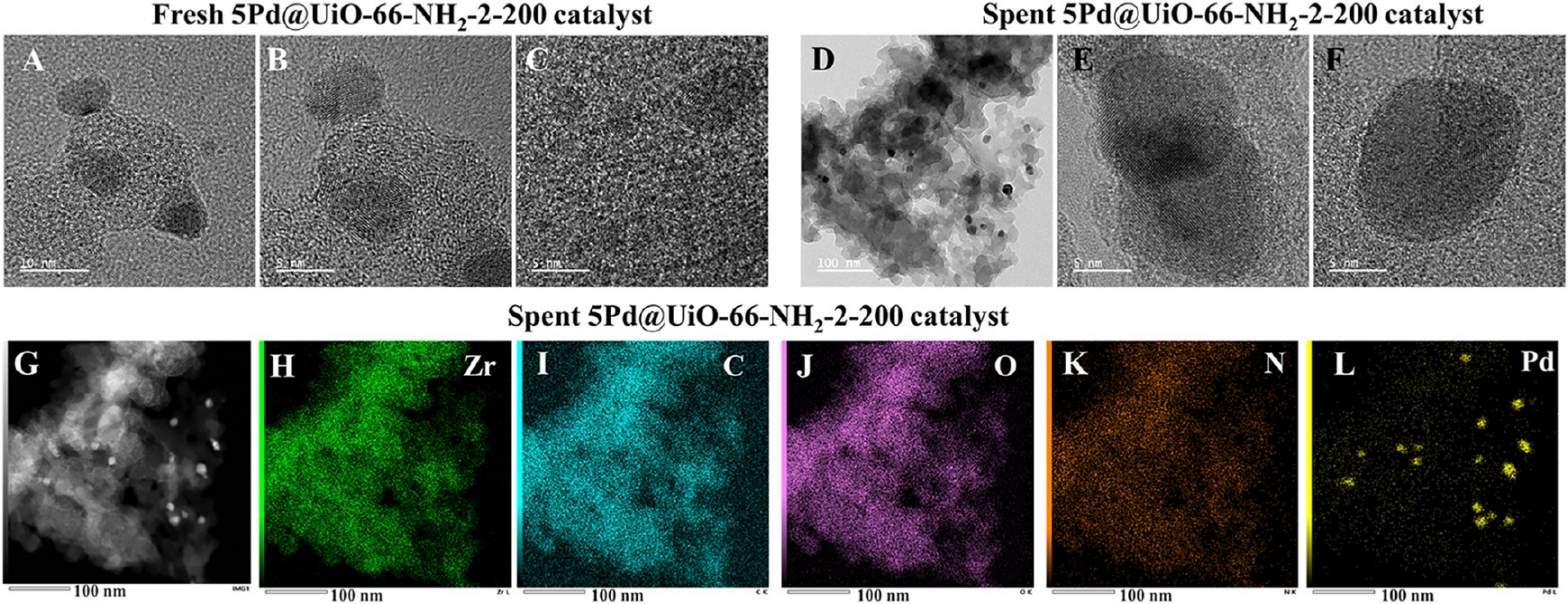
TEM and STEM-EDS characterization of the spent catalyst after the catalytic reaction. (A–F) TEM micrographs at different magnifications showing the morphology of the recovered material and the presence of enlarged Pd nanoparticles after reaction, (G) HAADF-STEM image and corresponding elemental mappings for (H) Zr, (I) C, (J) O, (K) N, and (L) Pd.

### Electrocatalytic Activity for HER

#### Linear Sweep Voltammetry

To evaluate the electrocatalytic activity of the MOFs for HER, a preliminary screening was performed using linear sweep voltammetry (LSV). The materials were suspended in ethanol at a concentration of 0.5 mg mL^–1^, and 10 μL of suspension were deposited by drop-casting onto a glassy carbon electrode (GCE). To evaluate the intrinsic activity of the materials without interference from polymer-catalyst interactions, no polymeric binder (e.g., Nafion or PTFE) was added to the formulations. The modified electrode was then used as the working electrode in a three-electrode electrochemical cell, featuring an Ag/AgCl reference electrode and a Pt wire counter electrode. The electrodes were immersed in a 0.5 M H_2_SO_4_ solution. The LSV were recorded over a potential range from 0.22 to −0.53 V vs RHE (which corresponds to 0 to −0.75 V vs Ag/AgCl), with a scan rate of 2 mV s^–1^ (Figure 12A). The current densities were calculated on the electrochemical surface area (ECSA), determined by capacitance measurements.^82–84^ At this stage, the performances of the electrocatalysts were primarily evaluated in terms of onset potential (i.e., the potential where an electrochemical reaction starts to occur),^85^ which was determined from the first derivative of the LSV (Figure S19).^86^ Moreover, another parameter commonly used to assess an electrocatalyst’s performance is the overpotential required to reach a current density of 10 mA cm^–2^. However, only 5Pd@UiO-66-NH_2_-2–200 reached that value of *j*, at an applied overpotential of −0.43 V vs RHE. To compare all other materials, we used an arbitrary parameter, the current density at −0.5 V (|*j*|_η=–0.5_). The performances were evaluated using the bare GCE as the negative control and a commercial catalyst based on Pd nanoparticles on carbon (5Pd@C) as the positive control. The voltammograms highlight the superior electrocatalytic activity of 5Pd@UiO-66-NH_2_-2–200, which outperforms all other materials, including the commercial 5Pd@C. Notably, the onset overpotential for HER with this material corresponds to ≈−2 mV vs RHE, and the value of |*j*|_η=–0.5_ is 13.94 mA cm^–2^. Other UiO-66-NH_2_-based materials did not show any catalytic activity, with values of current density comparable to the negative control. All other materials showed intermediate performances, with current densities between the negative and the positive control. The values of onset potential and |*j*|_η=–0.5_ for the best electrocatalysts are reported in Table 3, while the values for all materials are reported in the SI (Table S2).

**Figure 12:**
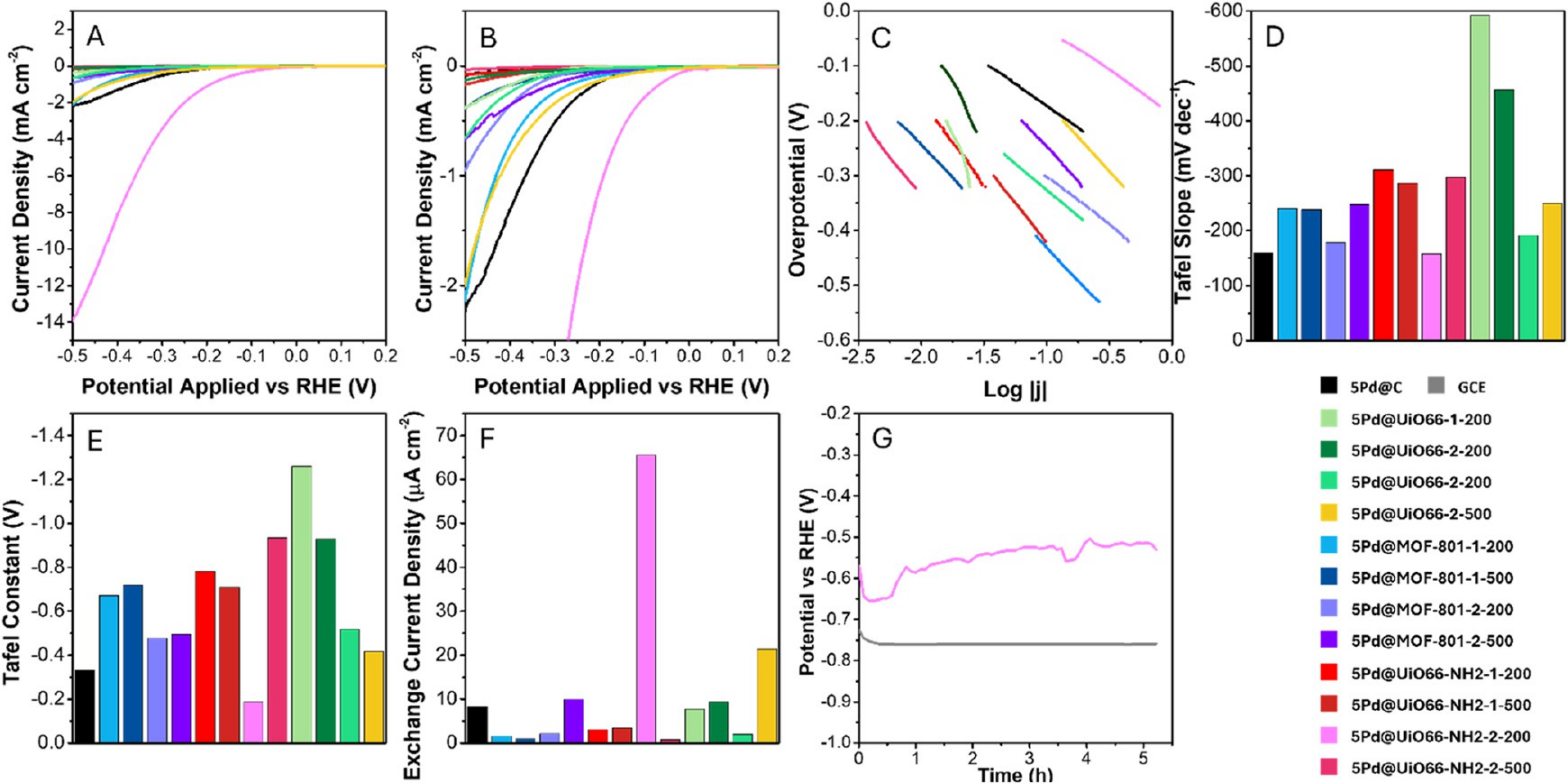
(A) Voltammograms recorded with all modified electrodes in H_2_SO_4_ 0,5 M at a scan rate of 2 mV/s; (B) magnification of the LSV; (C) Tafel plots for all the tested material; (D–F) histograms showing the values of Tafel slope (D), Tafel constant (E) and exchange current density (F); and (G) chronopotentiometry recorded with the electrode modified with 5Pd@UiO-66-NH_2_-2–200 vs the bare GCE.

**Table 3:** Onset Potential and |*j*|_η*=*–0.5_, Derived from LSV

material	onset potential (V)	|*j*|_η=–0.5_ (mA cm^–2^)
**5Pd@C**	–0.148	2.24
**5Pd@MOF-801–1–200**	–0.222	2.13
**5Pd@MOF-801–2–200**	–0.281	0.95
**5Pd@UiO-66-NH**_**2**_ **-2–200**	–0.002	13.94
**5Pd@UiO-66–2–500**	–0.117	2.00

#### Tafel Analysis

To have a better understanding and to quantify the electrocatalytic activity of these materials, the Tafel analysis was used to extract key kinetic parameters such as the Tafel slope (*b*), the Tafel constant (η_j=1_), and the exchange current density (*j*_0_).^87^ Tafel plots were obtained by plotting the overpotential (η) vs the log_10_ |*j*|, and isolating the region under the kinetic control regime for linear fitting (Figure 12B). The resulting equation is the Tafel equation^88^

$$\eta =b\log_{10}\left(j\right)-c$$

where η is the overpotential, *b* is the Tafel Slope, *j* is the current density, and *c* is a constant directly proportional to *j*_0_ (***c*** **=** ***b*** **log**_**10**_ **(** ***j***_**0**_ **))**.

The Tafel slope is a pivotal parameter to define the electrocatalytic properties of a material. It represents how much the overpotential must change to increase the reaction current by a factor of 10. Lower values of *b* are associated with better electrocatalytic performance.^89^ The Tafel constant can be obtained as the intercept of the Tafel plot and coincides with the overpotential that must be applied to deliver one unit of current density (***j*** **= 1**, ***η***_***j*** **=1**_ **=** ***b*** **log**_**10**_ **(** ***j***_**0**_ **)**).^90^ As for the Tafel slope, lower values of the Tafel constant indicate better electrocatalytic performances. Finally, the exchange current density is the current density theoretically recorded at the equilibrium potential (η = 0) and is a kinetic parameter directly proportional to the reaction rate constant. Consequently, higher values of *j*_0_ mean faster intrinsic kinetics.^91^ In Figure 12C–E, the three parameters are plotted as histograms. The corresponding values are reported in Table S2.

As can be seen from Figure 12C, the 5Pd@MOFs synthesized in two steps at 200 °C (5Pd@MOF-801–2–200, 5Pd@UiO-66-NH_2_-2–200, 5Pd@UiO-66–2–200) present the lowest values of *b*, comparable with that of the commercial 5Pd@C, outperforming the corresponding pyrolyzed materials (5Pd@MOF-801–2–500, 5Pd@UiO-66-NH_2_-2–500, 5Pd@UiO-66–2–500). The values of *b* for these materials are higher than the theoretical values.^92^ This could be related to the uncompensated resistance related to their low conductivity, which partially hinders the charge transfer. Conversely, Figure 12D clearly highlights the superior electrocatalytic performance of 5Pd@UiO-66-NH_2_-2–200 with respect to all other materials, displaying a value of η_*j*=1_ equal to −0.187 V, which is 1.8 times lower than that of 5Pd@C. Finally, Figure 12E shows that 5Pd@UiO-66-NH_2_-2–200 presents a *j*_0_ of 65.5 μA cm^–2^ that is 3 to 6 times higher than all other materials. From this evidence, it is possible to correlate the kinetic data for HER with the structural characterization of the materials and rationalize the obtained performances. All materials synthesized in two steps at 200 °C present lower values of *b* than their pyrolyzed counterpart and the materials synthesized in one step. However, when the overall performances of the 5Pd@MOF-2–200 are compared, only 5Pd@UiO-66-NH_2_-2–200 emerges as a suitable candidate for the HER catalyst. From the STEM-EDX images (Figures 5 and S7–S9), it is evident that this material is characterized by the presence of homogeneously dispersed small Pd nanoparticles on the surface, which leads to the maximization of active sites available for the adsorption of H^+^ during the first step (Volmer step) of HER. Moreover, the presence of amine functionalities (−NH_2_) within its structure not only stabilizes the Pd nanoparticles but also provides an electron-rich microenvironment, enhancing the intrinsic kinetic rate (*j*_0_) with respect to the materials based on fumaric and terephthalic acid.

Interestingly, 5Pd@MOF-801–1–200, which showed a low onset potential in the LSV, exhibits a high *b* and η_*j*=1_, and a low *j*_0_, indicating slow kinetics. This observation clearly demonstrates the importance of performing the comprehensive Tafel analysis to define the overall performance of an electrocatalyst, as the onset potential alone is not a sufficient parameter to assess a material’s intrinsic electrocatalytic activity and stability under working conditions.

Finally, the durability of 5Pd@UiO-66-NH_2_-2–200 was tested by chronopotentiometry at a constant current density of 4 mA cm^–2^ for 5.5 h.^93^ As can be observed in Figure 12F, upon the first 45 min, it slowly shifted to a more positive potential, indicating a gradual activation of the material. This behavior could be related to an activation process that removes oxides through the application of the cathodic current. Then, the potential remained almost constant (≈−0.5 V) during the experiment.

### Literature Comparison for HER

To properly contextualize the electrocatalytic efficiency of the extruded Pd@MOF materials, we investigated their fundamental kinetic parameters, the *j*_0_, and the Tafel slope, and compared our synthetic approach with recent literature. For our optimized 5Pd@UiO-66-NH_2_-2–200, we observed a notable *j*_0_ of 65.5 μA cm^–2^, indicating a well-defined intrinsic rate of electron transfer at the catalyst–electrolyte interface. The kinetic performance was further evaluated via its Tafel slope (158 mV dec^–1^). A Tafel slope of this magnitude suggests that the Volmer step (the initial adsorption of protons) is the rate-determining step.^94,95^ This behavior is highly characteristic of unpyrolyzed, highly porous frameworks, where the inherently low electrical conductivity of native MOFs strictly limits charge transfer.^96,97^

To overcome this critical kinetic bottleneck without resorting to destructive high-temperature carbonization (which leads to Pd sintering and the loss of stabilizing –NH_2_ groups), literature strategies typically rely on complex, multistep hybridization protocols to artificially lower the Tafel slope.^94,98^ For instance, highly conductive carbonaceous materials are frequently mixed or intercalated within the MOFs to create conductive networks. Makhafola et al. reported a Tafel slope of 158 mV dec^–1^ for a palladinized metal–organic framework, but only after the deliberate addition of conductive graphene oxide sheets (Pd@GO/MOF) via a multistep impregnation method.^95^ Similarly, the incorporation of highly expensive single-walled carbon nanotubes (SWCNTs) into an MOF-199 matrix via hydrothermal routes was necessary to achieve a Tafel slope of 172 mV dec^–1^.^99^ In another composite approach, Monama et al. utilized electroactive organic dyes, blending copper phthalocyanines with the MOF to serve as a conductive support for Pd electrochemical deposition, which yielded a Tafel value of 177 mV dec^–1^.^94^ Other recent systems aim to bypass carbon additives by designing intrinsically conductive frameworks, although at a higher synthetic cost. For example, Wang et al. synthesized Pd-loaded metal-hexaaminotriphenylene MOFs (M-HITP, where M = Fe, Ni, Cu, Zn), relying on expensive conductive linkers and multistep synthesis. Depending on the metal node used, they reached Tafel slopes ranging between 73 and 229 mV dec^–1^. Likewise, Yue et al. developed vinylene-linked covalent organic frameworks (PY-SE-COF-Pd) requiring waste-generating steps and complex Knoevenagel condensation reactions to report a Tafel slope of 150 mV dec^–1^.^98^

While all these approaches are effective in enhancing electrocatalytic kinetics, resulting in Tafel slopes comparable to or slightly lower than our 158 mV dec^–1^, they heavily rely on toxic additives, expensive organic linkers, and multistep solvothermal procedures. In stark contrast, the 5Pd@UiO-66-NH_2_-2–200 catalyst reaches highly competitive kinetic parameters through a single-step, solvent-free mechanochemical approach. Strikingly, the Tafel slope of our extruded material perfectly matches, and in some cases outperforms, the kinetics of other complex multicomponent systems, completely bypassing the need for graphene, carbon nanotubes, or complex polymeric binders. This clearly demonstrates that mechanochemical extrusion induces a beneficial degree of framework amorphization. These extrusion-induced structural defects effectively mimic the role of conductive additives, facilitating the mass and charge transport. Ultimately, this validates continuous-flow mechanochemistry as a superior, green, and highly scalable alternative for the design of functional, noncrystalline MOF electrocatalysts.

## Experimental Section

### Reagents and Materials

All chemicals used for the synthesis of catalysts and during catalytic reactions were commercially available (Sigma-Aldrich) and used as received, unless otherwise specified. A complete list of reagents and their specifications is reported in Table S1.

#### Synthesis of Acetate Cluster [Zr_6_O_4_(OH)_4_(CH_3_COO)_12_]_2_

The acetate cluster [Zr_6_O_4_(OH)_4_(CH_3_COO)_12_]_2_ (**1**) was synthesized following the procedure reported in the literature.^100^ Zirconium­(IV) propoxide (70 wt % in 1-propanol) (1 g, 3.05 mmol) and acetic acid (3.50 mL) (1:20 molar ratio) were mixed in a sealed beaker and left to react overnight at room temperature. The resulting microcrystalline colorless solid was collected by suction filtration, gently washed with acetic acid, and dried at room temperature.

#### Synthesis of MOF Catalytic Materials

MOF-based catalytic materials were prepared by reactive extrusion using a ZE 12 HMI twin-screw extruder (Three Tec, Seon, Switzerland). Three amorphous MOF supports were produced under solvent-free conditions: UiO-66, UiO-66-NH_2_, and MOF-801.

The synthesis of UiO-66 was performed using Zr_12_ cluster 1 (3 g, 1.065 mmol), terephthalic acid (2.15 g, 12.773 mmol, 12 equiv), and Et_3_N (3 mL). The mixture was extruded at 40 °C and 180 rpm for 2 h, following a previously reported method.^36^ The product was collected using a small amount of ethanol and dried under vacuum at 70 °C overnight, affording 2.57 g of material (86% yield) based on Zr_12_ cluster **1**.

UiO-66-NH_2_ and MOF-801 were synthesized by using the same general procedure, substituting terephthalic acid with 2-aminoterephthalic acid (2.50 g, 12.775 mmol) or fumaric acid (1.483 g, 12.775 mmol), respectively. For UiO-66-NH_2_, Et_3_N was omitted because of the presence of the amino substituent.

#### Synthesis of Pd@MOF Materials

##### One-Step Process (5Pd@MOF-1–200)

In the one-step approach, the palladium precursor was incorporated directly into the solid reaction mixture, enabling in situ formation of Pd nanoparticles during extrusion. The extruder was charged with 1 g of Zr_12_ cluster 1, 12 equiv of the appropriate organic linker (terephthalic acid for UiO-66, 2-aminoterephthalic acid for UiO-66-NH_2_, and fumaric acid for MOF-801), Pd­(OAc)_2_ (5 wt % Pd target loading), 1.0 mL of ethylene glycol, and, except for the amino-functionalized system, 1.0 mL of Et_3_N. Extrusion was carried out at 190 °C and 180 rpm for 2 h, promoting framework formation and palladium deposition on the surface.

##### Two-Step Process (5Pd@MOF-2–200)

In the two-step approach, the palladium precursor was added after the extrusion-assisted synthesis of the amorphous MOF, enabling postsynthetic incorporation under mild thermal conditions. Ethylene glycol (1.0 mL) was again employed as a reducing agent. The extrusion temperature was set to 200 °C, with a screw speed of 50 rpm and a total processing time of approximately 30 min.

After processing, all materials were washed with ethanol to remove residual unreacted species and dried under vacuum at 70 °C overnight.

#### Synthesis of Pd@MOF Thermally Treated Samples

Thermal treatments were performed in a tubular furnace under an N_2_ atmosphere at 500 °C for 1 h, using a 5 °C min^–1^ heating rate. The resulting materials were designated as 5Pd@MOF-1–500 and 5Pd@MOF-2–500, respectively.

### Characterization Techniques

Powder X-ray diffraction (XRD) patterns were collected on a Bruker D8 Advance diffractometer using Cu Kα radiation and a LynxEye detector (2θ = 8–80°, scan rate = 0.08° min^–1^).

X-ray photoelectron spectroscopy (XPS) analyses were carried out using a Physical Electronics VersaProbe II Scanning XPS Microprobe with a monochromatic Al Kα source under 10^–7^ Pa. Spectra were calibrated to the C1s peak at 284.6 eV, with data fitting performed using PHI ACCESS ESCA-F V6 software (Shirley background and Gauss–Lorentz profiles).

N_2_ physisorption measurements were recorded at −196 °C using a Micromeritics ASAP 2000 instrument. Samples were outgassed at 120 °C for 2 h. Surface areas were calculated using the BET method, while pore size distributions were derived via the BJH model using Micromeritics software.

Scanning electron microscopy with energy-dispersive X-ray (SEM-EDX) mapping was performed using a JEOL JSM-7800 LV microscope. Transmission electron microscopy (TEM) was conducted on a JEOL 2010 and FEI Tecnai G2 system operating at 200 kV, equipped with a CCD camera. Samples were dispersed in ethanol and deposited on holey-carbon-coated 300 mesh Cu grids.

Palladium content was quantified by inductively coupled plasma optical emission spectroscopy (ICP-OES) using an Avio 550 Max instrument.

### Catalytic Experiments

#### Suzuki–Miyaura Cross-Coupling Reaction

Catalytic activity was evaluated in the Suzuki–Miyaura reaction using iodobenzene (0.25 mmol, 26 μL), phenylboronic acid (0.35 mmol, 43 μL), and K_2_CO_3_ (0.5 mmol, 70 mg) in ethanol (3 mL). As a first approximation, the reaction mixture, containing 10 mg of catalyst, was sealed and stirred at 80 °C for 4 h. Further parametric analysis was performed, leading to 60 °C and 40 min as optimized reaction conditions. After completion, the catalyst was separated by filtration. Conversions and selectivities were determined by GC–FID (Agilent 6890 N, HP-5 capillary column, N_2_ flow = 60 mL min^–1^, pressure = 20 psi), while product identification was confirmed by GC-MS (Agilent 7820 A GC/5977B HES MSD) and ^1^H NMR in the Bruker Avance III HD 400 WB equipped with a 4 mm CP/MAS probe, employing deuterated chloroform as solvent.

#### Electrocatalytic HER

Electrocatalytic tests were carried out in a conventional three-electrode cell using a platinum wire as the counter electrode, Ag/AgCl (3 M KCl) as the reference, and a glassy carbon electrode (diameter = 0.3 cm) coated with the catalyst as the working electrode, using a Metrohm Autolab PGSTAT30 potentiostat interfaced to a PC with Nova 2.1.8 software. Before each experiment, the GCE was first cleaned with methanol and then polished on alumina 0.2 μm and sonicated in Milli-Q water, and was then modified by drop-casting 10 μL of 0.5 mg/mL suspensions of the materials. Cyclic voltammograms (CV) were recorded at different scan rates (10, 20, 40, 60, 80, 100, 150, 200, 250, 300 mV s^–1^), in 0.5 M H_2_SO_4_ to determine the electrochemical surface area (ECSA). The capacitive current was sampled at 0.2 V vs Ag/AgCl. Linear sweep voltammetry (LSV) curves were recorded at a scan rate of 2 mV s^–1^. Tafel slopes were derived from the corresponding LSV data. The analysis of electrochemical data was carried out with Origin and Microsoft Excel.

## Conclusions

This work demonstrates that extrusion-based mechanochemistry is a powerful and sustainable platform for the synthesis of palladium-modified metal–organic frameworks. Using a continuous-flow extrusion process, three Zr-based MOFs, MOFs-UiO-66, UiO-66-NH_2_, and MOF-801, were directly synthesized and functionalized with palladium nanoparticles, yielding amorphous Pd@MOF materials containing highly accessible, quasi-spherical Pd species. The chemical nature of the parent linkers proved to be a decisive factor in governing metal dispersion, with UiO-66-NH_2_ exhibiting the highest ability to stabilize small and homogeneously distributed Pd nanoparticles. This highlights the importance of functional group engineering in mechanochemical catalyst design.

The catalytic evaluation of the extruded materials in two distinct applications: (i) carbon–carbon bond formation and (ii) electrocatalytic hydrogen production, further underscores their versatility. In cross-coupling reactions, the Pd@MOFs delivered competitive or superior performance compared to conventional heterogeneous catalysts, particularly after optimization of reaction parameters with a sustainability-oriented approach. Their ability to efficiently activate both aryl bromides and the more challenging aryl chlorides confirms the robustness of the active Pd sites generated during extrusion. Recycling studies validated the operational stability of the catalysts, indicating that the extruded amorphous MOF matrices withstand multiple reaction cycles without significant structural degradation.

In the hydrogen evolution reaction, the materials also demonstrated a promising electrocatalytic activity. The finely dispersed Pd nanoparticles provided a high density of active sites for proton reduction, while the amine-functionalized UiO-66-NH_2_ support enhanced the Pd stabilization and created an electron-rich microenvironment that improved the intrinsic kinetic rate. Among the series, 5Pd@UiO-66-NH_2_-2–200 emerged as the most active HER catalyst.

The results of this study open several avenues for future research. A deeper mechanistic understanding of the extrusion synthesis process could clarify how process parameters influence the MOF formation and Pd nanoparticle dispersion. In addition, extending this methodology to other metals or bimetallic systems could unlock additional synergistic effects for both synthetic and energy-related applications.

## Supplementary Material



## Notes

Materials characterization, Hammett plot analysis, TOF calculation, CHEM21 metrics calculations, and electrocatalytic HER data (PDF)

## Abbreviations

HER: hydrogen evolution reaction
MOFs: metal–organic frameworks
XRD: X-ray diffraction
XPS: X-ray photoelectron spectroscopy
HR-TEM: high-resolution transmission electron microscopy
SEM-EDX: scanning electron microscopy with energy-dispersive X-ray
ICP-OES: inductively coupled plasma optical emission spectroscopy
PMI: process mass intensity
RME: reaction mass efficiency
AE: atom economy
OE: overall efficiency
LSV: linear sweep voltammetry
CV: cyclic voltammograms
GCE: glassy carbon electrode
ECSA: electrochemical surface area
