# Supplementary material for: Pd-Modified Metal Organic Frameworks Synthesized via Mechanochemical Extrusion: Versatile Materials for Suzuki–Miyaura Cross-Coupling and Electrochemical Hydrogen Evolution Reaction
Source: ACS Sustain Chem Eng. 2026 Jun 9;14(24):11165–82. doi: 10.1021/acssuschemeng.6c03049 (PMC13293022; doi:10.1021/acssuschemeng.6c03049)
Supplement: Supplementary file 1 [file sc6c03049_si_001.pdf]

Pd-modified Metal Organic Frameworks synthesized via mechanochemical extrusion: versatile materials for Suzuki-Miyaura cross-coupling and electrochemical hydrogen evolution reaction

Paola Monaco,†‡ Oscar Trentin,†‡ Daniel Ballesteros-Plata,†† Giuseppe Misia,† Alessandro Silvestri,† Enrique Rodríguez-Castellón,†† Maurizio Selva,† Alvise Perosa,† Daily Rodríguez-Padrón, †,†††**

† Department of Molecular Science and Nanosystems, Ca' Foscari University of Venice, Via Torino 155, 30175, Venezia Mestre, Italy. A.P. alvise@unive.it

†† Department of Inorganic Chemistry, Facultad de Ciencias, Instituto Interuniversitario de Investigación en Biorrefinerías I3B, Universidad de Málaga, Campus de Teatinos s/n, 29071 Málaga, Spain.

††† Section of Chemistry for the Technology (ChemTech), Department of Industrial Engineering, University of Padova, Via Marzolo 9, 35131, Padova (PD), Italy. D.R.P daily.rodriguezpadron@unipd.it

‡ These authors contributed equally.

ABSTRACT

Extrusion-based mechanochemistry offers a sustainable route for materials synthesis. Here, we report the preparation of Pd-modified Metal-Organic Frameworks (UiO-66, UiO-66-NH_2, and MOF-801) via continuous-flow twin-screw extrusion. The resulting amorphous Pd@MOF materials contain quasi-spherical Pd nanoparticles whose dispersion is strongly influenced by the functional groups of the parent MOFs, with UiO-66-NH_2 providing superior stabilization. The catalysts were evaluated in

two applications: the Suzuki–Miyaura cross-coupling reaction and the electrochemical hydrogen evolution reaction (HER). Optimization of the Suzuki–Miyaura conditions afforded improved temperature and time parameters compared to literature examples, delivering higher TOFs and meeting CHEM21 First Pass sustainability criteria. A substrate scope analysis, including aryl bromides and chlorides, further confirmed their efficiency, while recycling studies demonstrated catalyst stability. In HER, the highly dispersed Pd nanoparticles maximized active-site availability, with 5Pd@UiO-66-NH_2-2-200 showing the best performance. The presence of amino groups in the UiO-66-NH_2 support provided an electron-rich environment that enhanced the intrinsic kinetic rate (j_0). Overall, extrusion mechanochemistry enables robust, versatile Pd@MOF catalysts for both organic synthesis and energy-related applications.

TABLE OF CONTENTS

MATERIALS CHARACTERIZATION 3

CATALYTIC ACTIVITY 10

Hammet plot analysis 10

TOF calculation 11

CHEM21 Metrics Calculations 13

Electrocatalytic HER 15

NMR AND MASS SPECTRA 19

MATERIALS CHARACTERIZATION

Table S1. Textural properties of the obtained catalytic materials

Sample	S_{BET} (m^2/g)	V_{BJH} (cm^3/g)	D_{BJH} (nm)
**UiO-66**	498	0.3	2.66
**5Pd@UiO-66-2-200**	319	0.3	3.88
**5Pd@UiO-66-2-500**	76	0.1	7.34
**5Pd@UiO-66-1-200**	214	0.3	4.90
**UiO-66-NH_2**	173	0.3	6.55
**5Pd@UiO-66-NH_2-2-200**	104	0.2	7.48
**5Pd@UiO-66-NH_2-2-500**	62	0.2	10.27
**5Pd@UiO-66-NH_2-1-200**	197	0.2	3.60
**MOF-801**	293	0.2	3.08
**5Pd@MOF-801-2-200**	227	0.02	0.42
**5Pd@MOF-801-2-500**	71	0.1	3.61
**5Pd@MOF-801-1-200**	34	0.1	10.71

* S_{BET}: specific surface area was calculated using the Brunauer-Emmett-Teller (BET) equation. V_{BJH}: pore volumes were calculated using the Barret-Joyner-Halenda (BJH) equation.D_{BJH}: mean pore size diameter was calculated using the BarretJoyner-Halenda (BJH) equation.

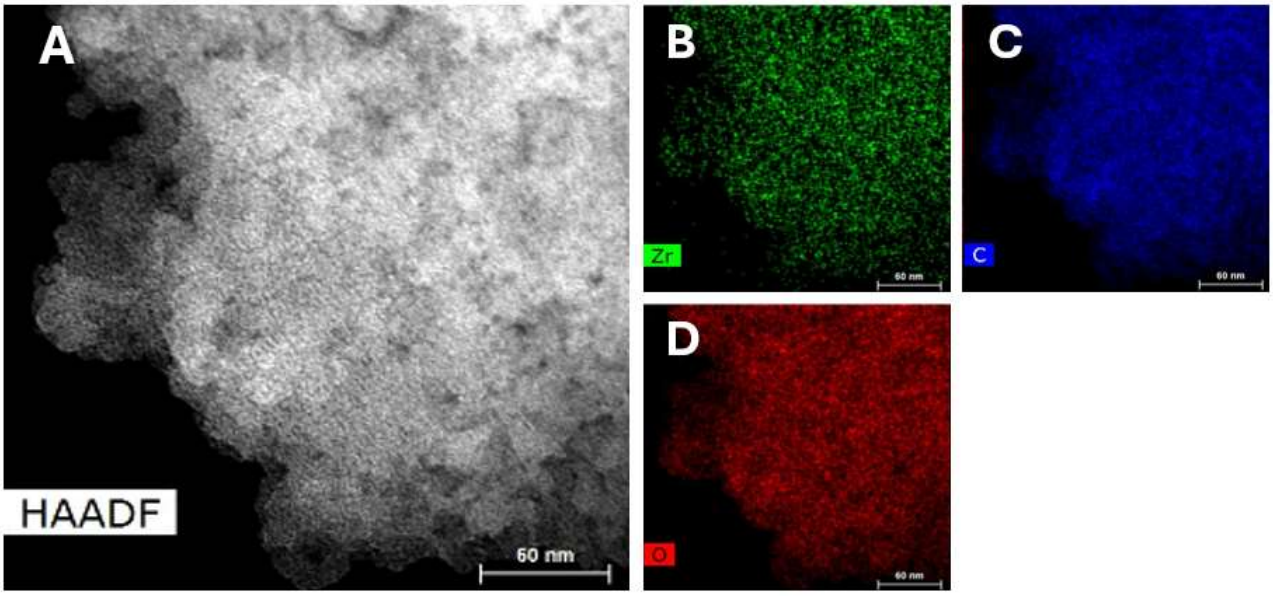

Figure S1: EDX mapping and STEM micrographs of **UiO-66** material. STEM images of UiO-66 (A). EDX-mapping results of UiO-66 for zirconium (B), carbonium (C) and oxygen (D).

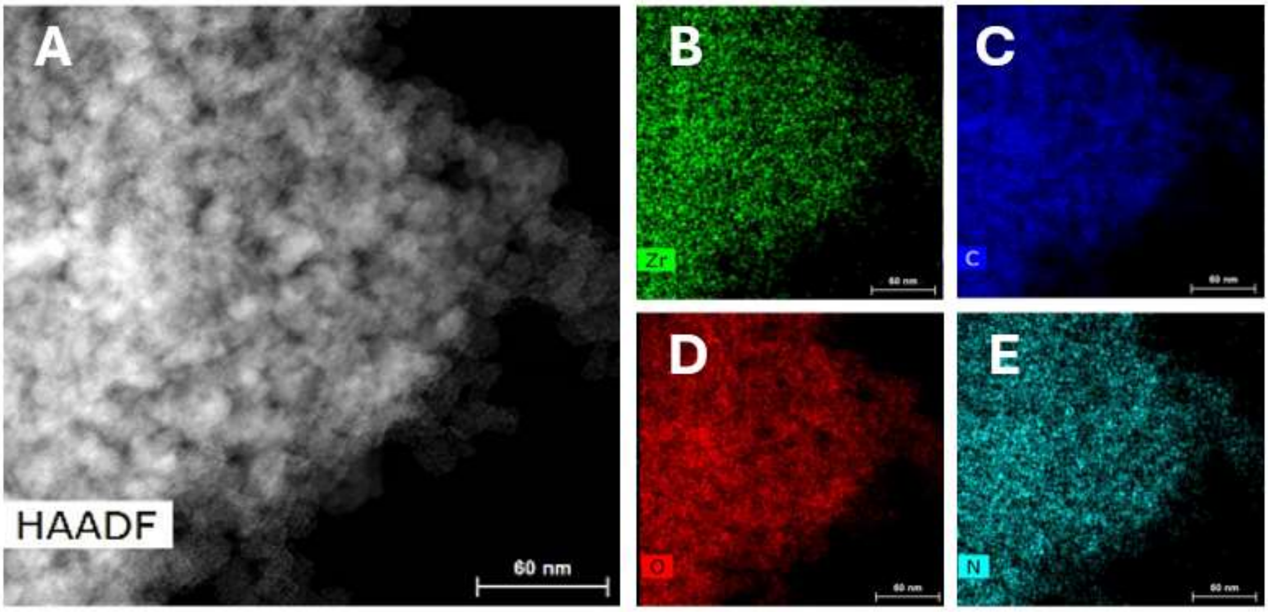

Figure S2: EDX mapping and STEM micrographs of **UiO-66-NH$_2$** material. STEM images of UiO-66-NH$_2$ (A). EDX-mapping results of UiO-66-NH$_2$ for zirconium (B), carbonium (C), oxygen (D) and nitrogen (E).

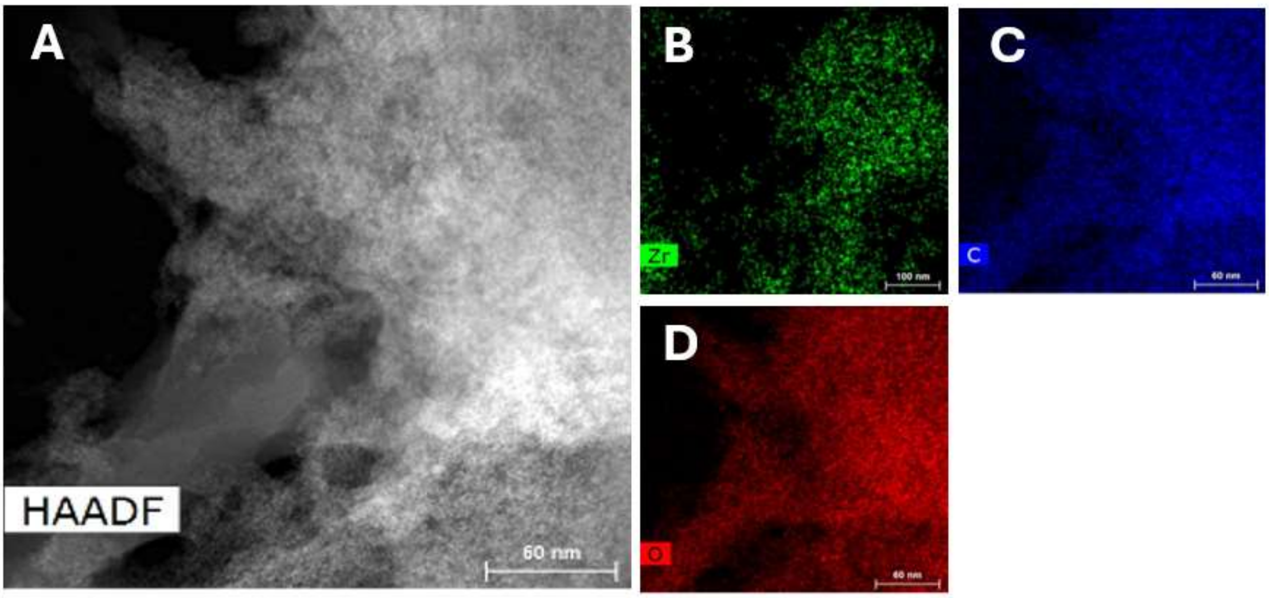

Figure S3: EDX mapping and STEM micrographs of **MOF-801** material. STEM images of MOF-801 (A). EDX-mapping results of MOF-801 for zirconium (B), carbonium (C) and oxygen (D).

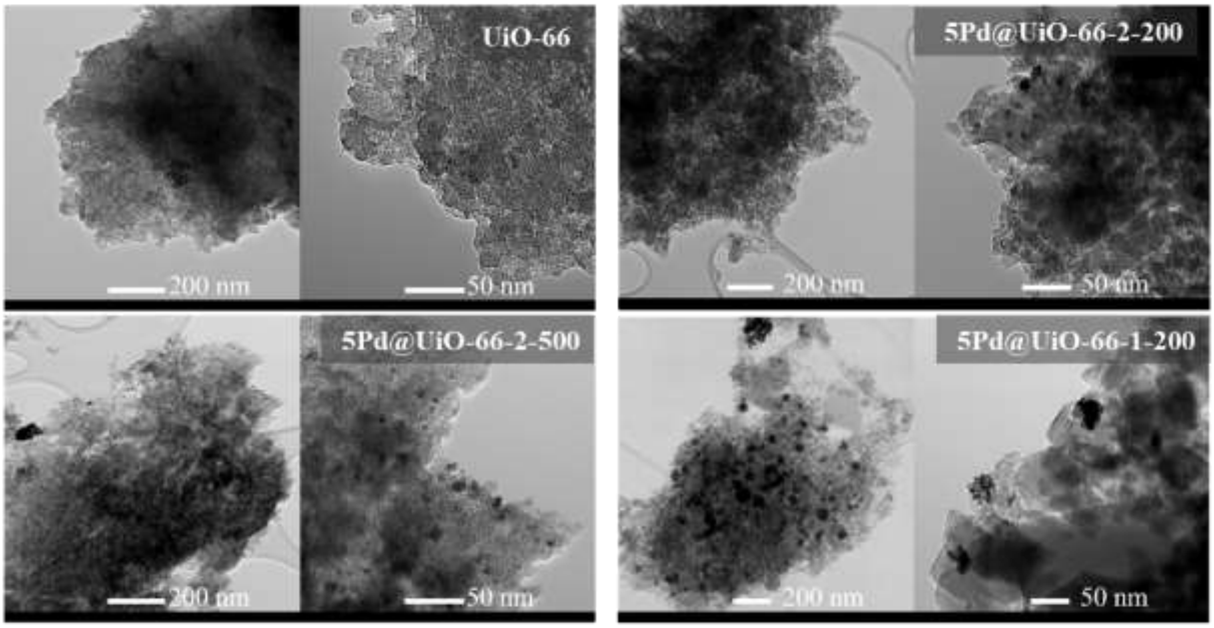

Figure S4. HR-TEM micrographs of bare **UiO-66** sample and after addition of Pd.

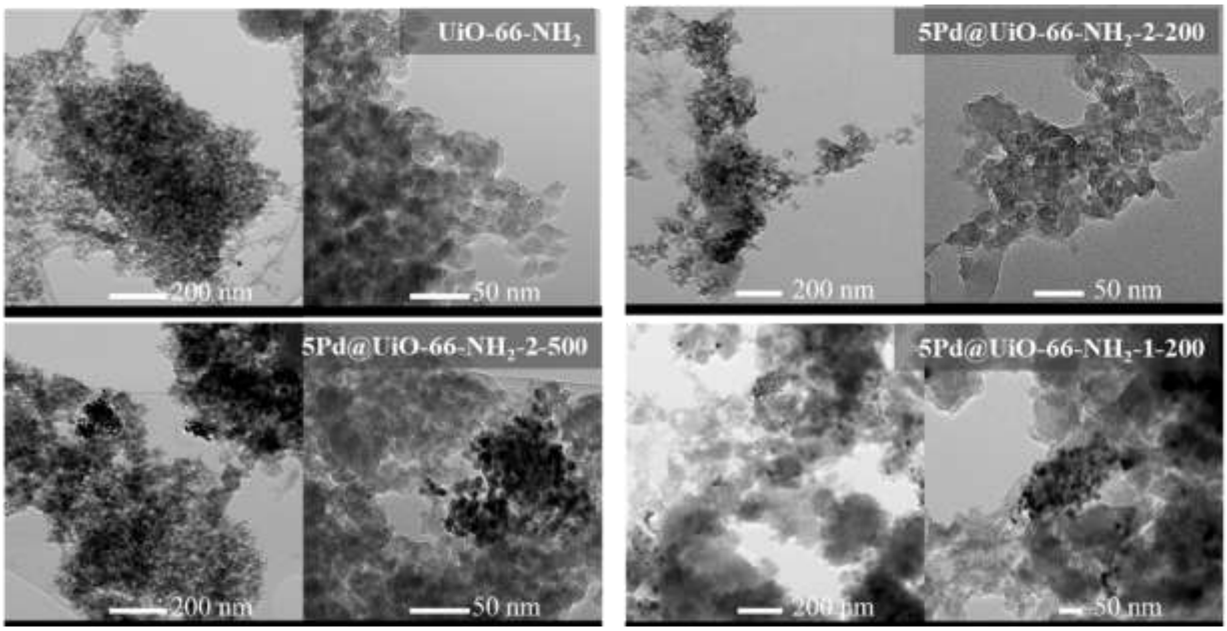

Figure S5. HR-TEM micrographs of bare **UiO-66-NH$_2$** sample and after addition of Pd.

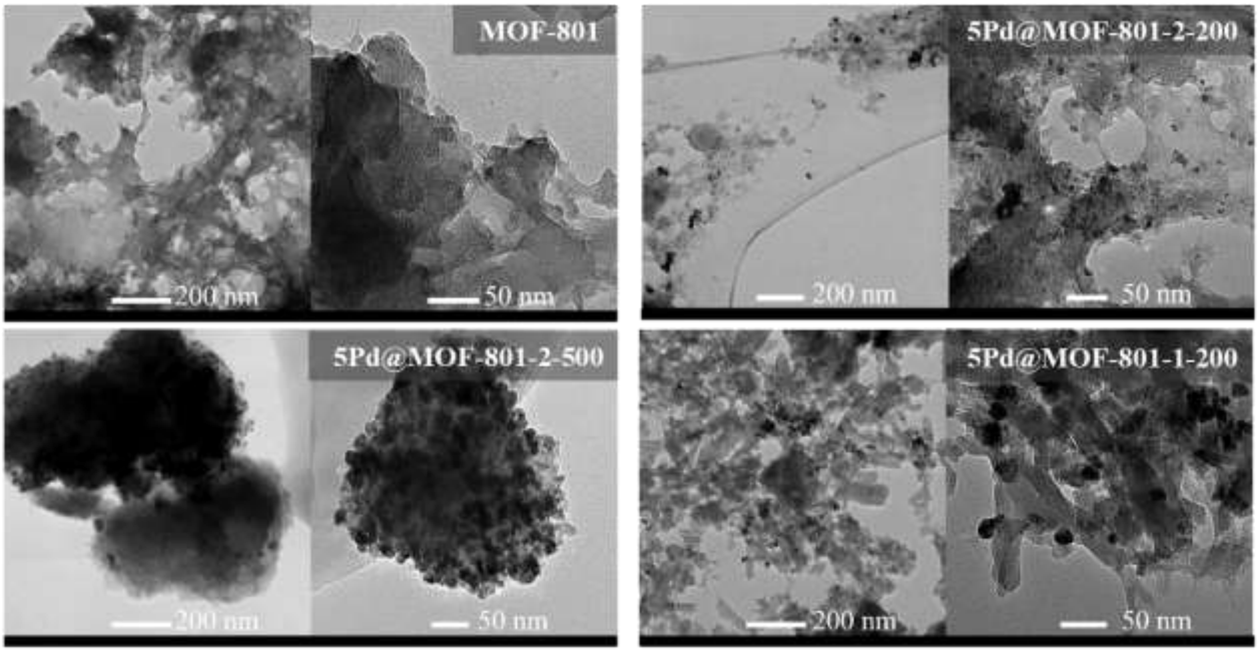

Figure S6. HR-TEM micrographs of bare **MOF-801** sample and after addition of Pd.

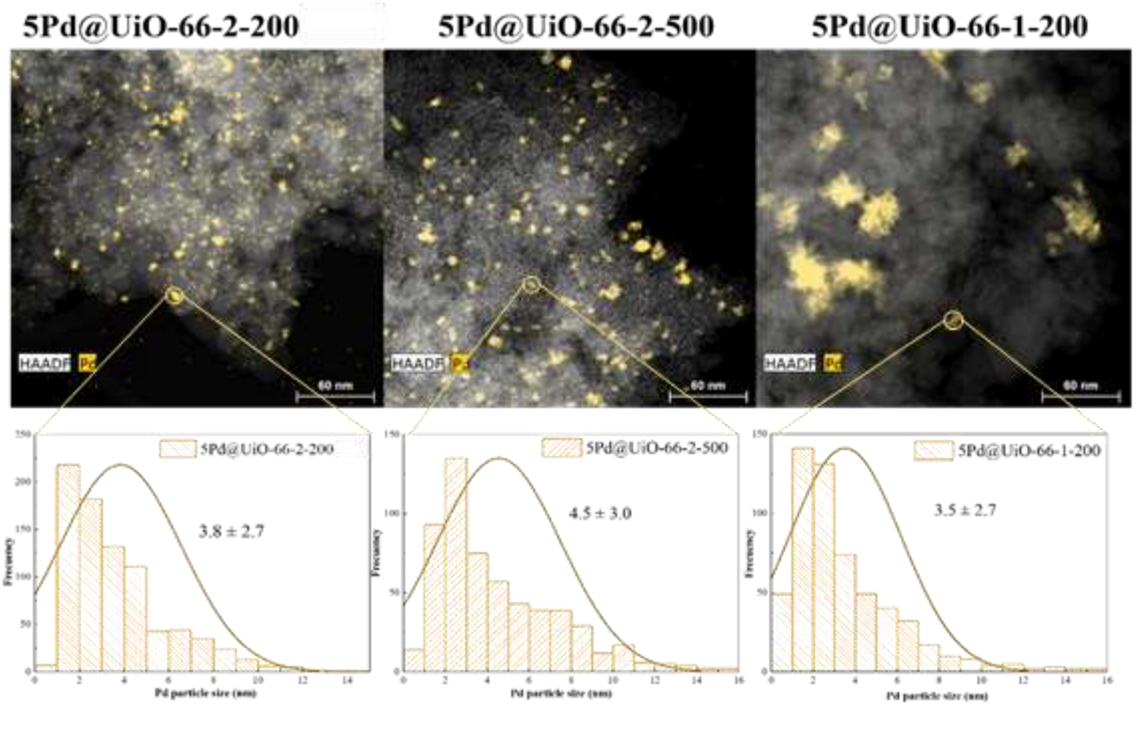

Figure S7. EDX of palladium particles in **UiO-66** material and their respective particle size distribution diagrams.

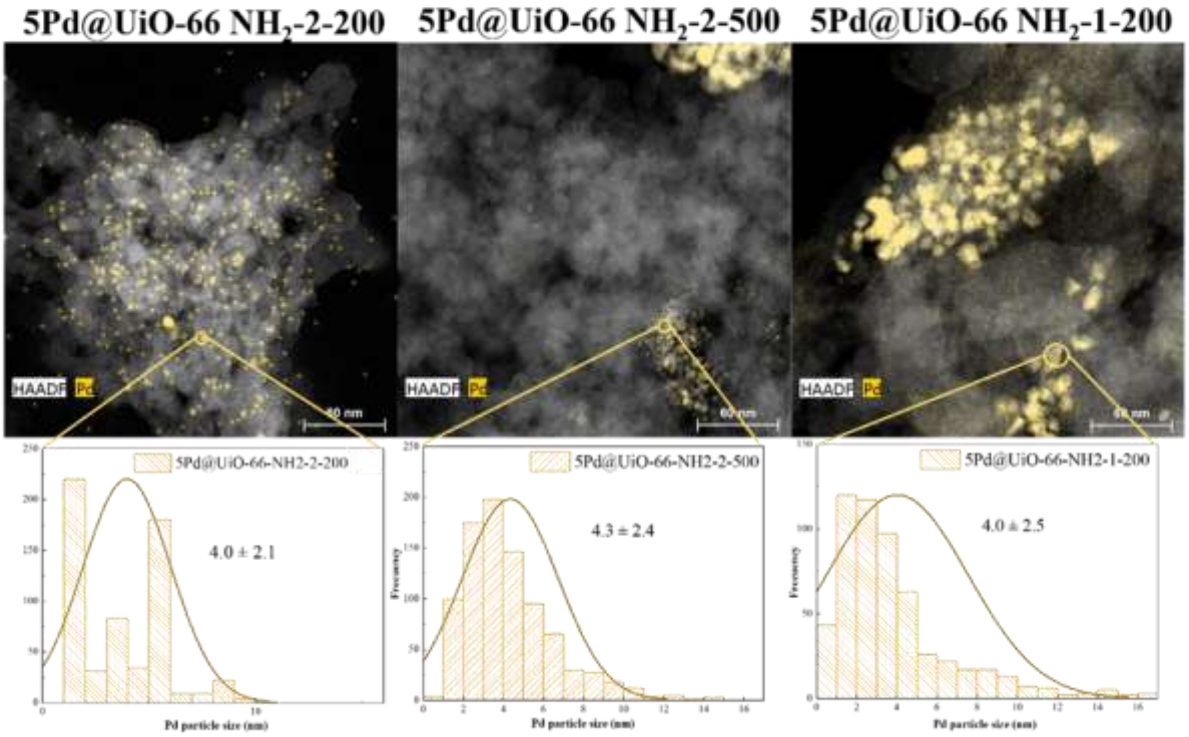

Figure S8. EDX of palladium particles in UiO-66-NH_2 material and their respective particle size distribution diagrams.

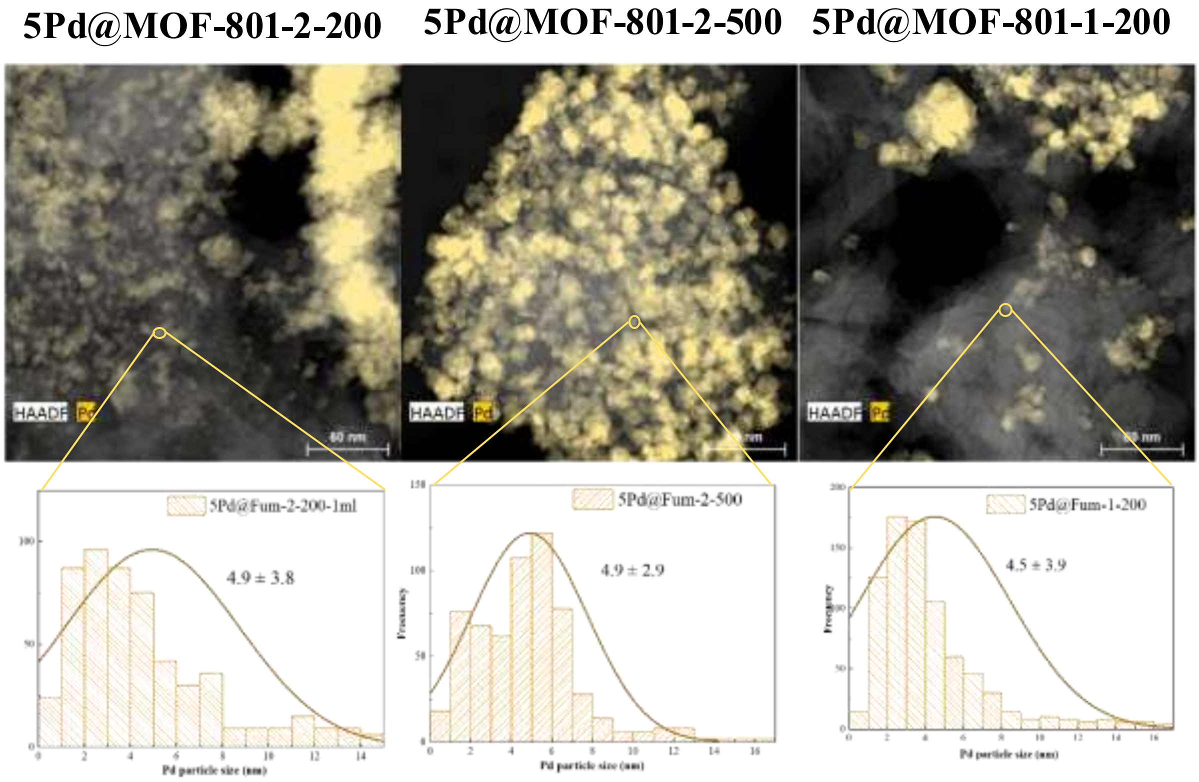

Figure S9. EDX of palladium particles in **MOF-801** material and their respective particle size distribution diagrams.

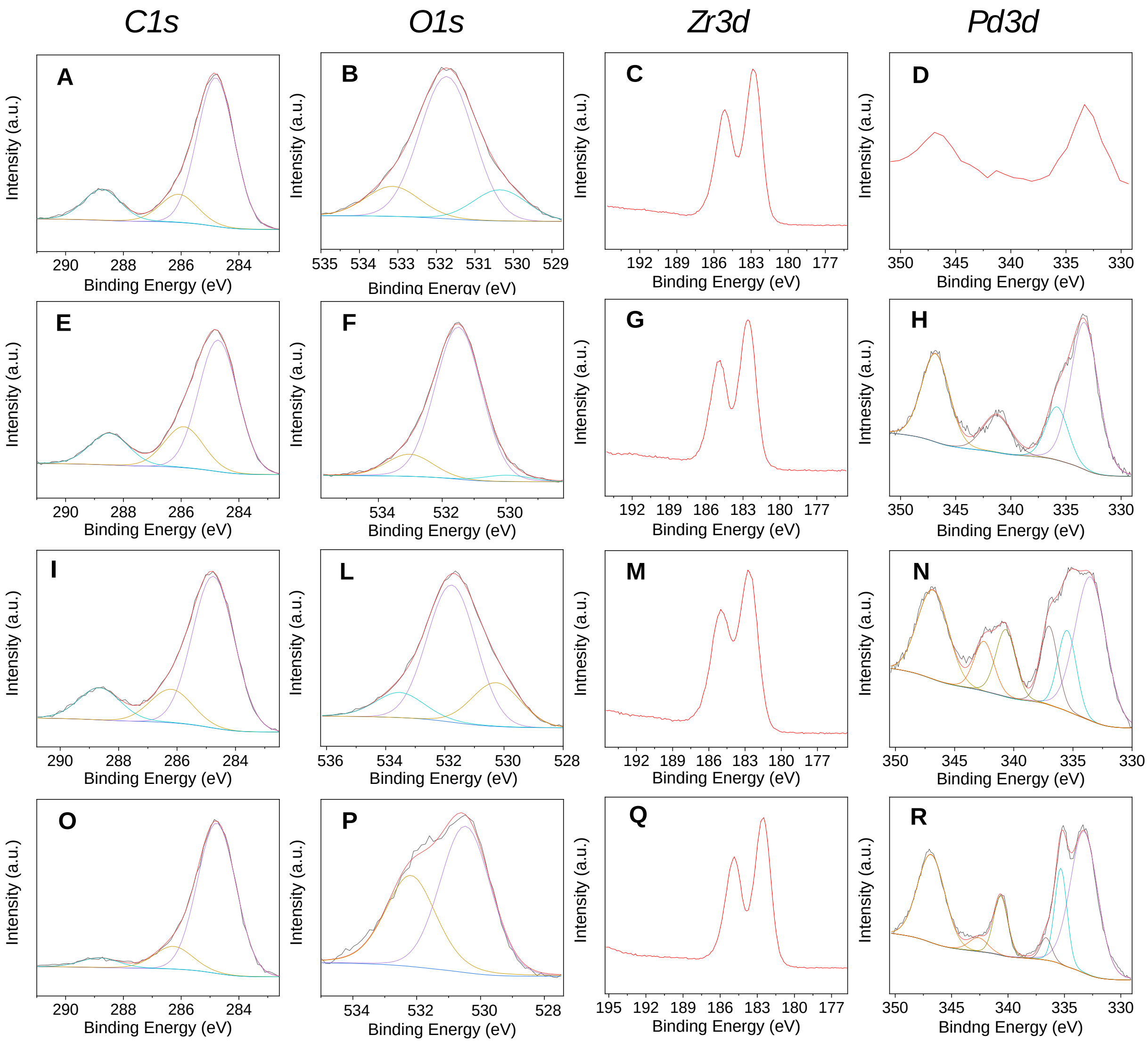

Figure S10: XPS spectra of **UiO-66** (A-D), **5Pd@UiO-66-1-200** (E-H), **5Pd@UiO-66-2-200** (I-N), **5Pd@UiO-66-2-500** (O-R). For each catalytic system, it is represented in this order the C1*s* (A, E, I, O), O1*s* (B, F, L, P), Zr3*d* (C, G, M, Q) and Pd3*d* (D, H, N, R) XPS regions.

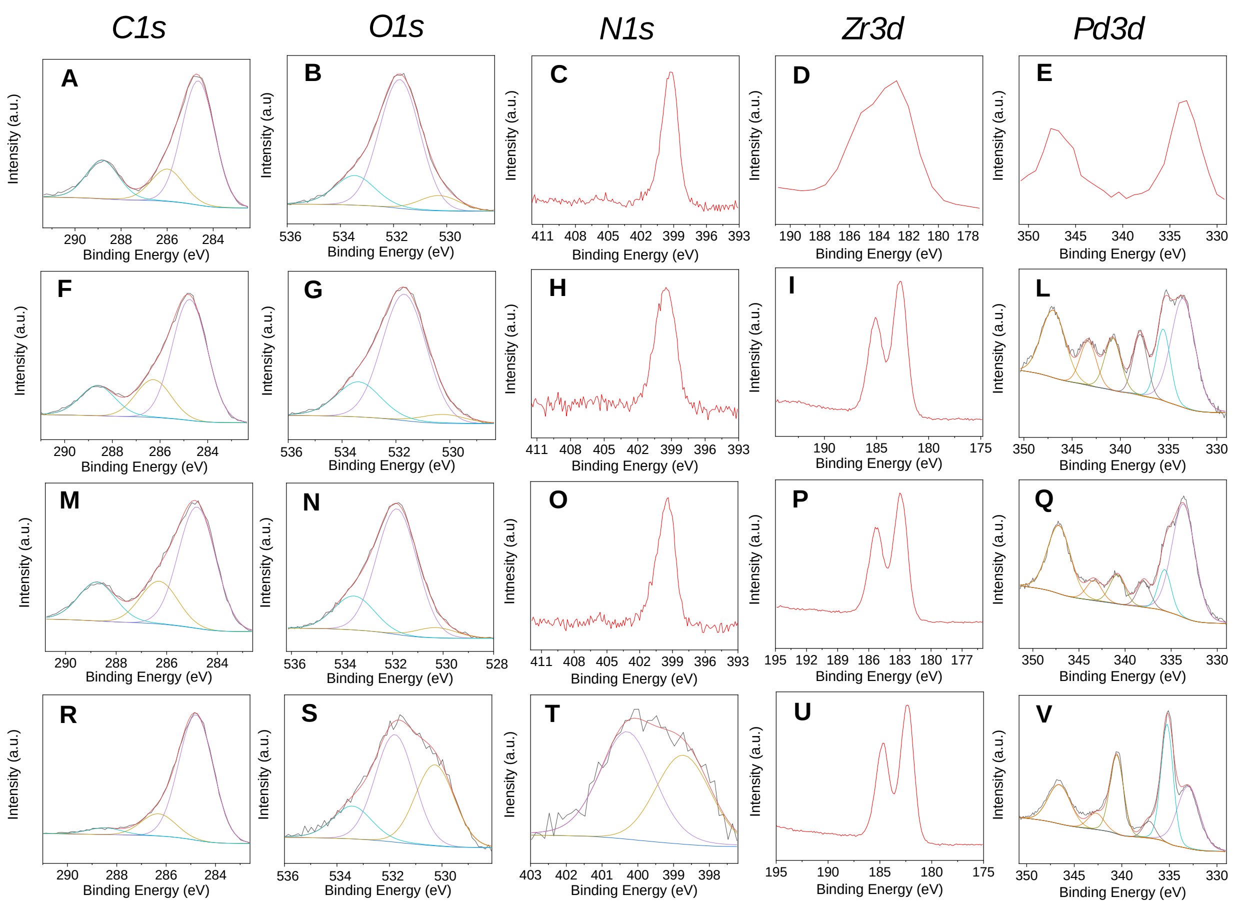

Figure S11: XPS spectra of **UiO-66-NH₂** (A-E), **5Pd@UiO-66-NH₂-1-200** (F-L), **5Pd@UiO-66-NH₂-2-200** (M-Q), **5Pd@UiO-66-NH₂-2-500** (R-V). For each catalytic system, it is represented in this order the C1*s* (A, F, M, R), O1*s* (B, G, N, S), Zr3*d* (C, H, O, T), N1s (D, I, P, U) and Pd3*d* (E, L, Q, V) XPS regions.

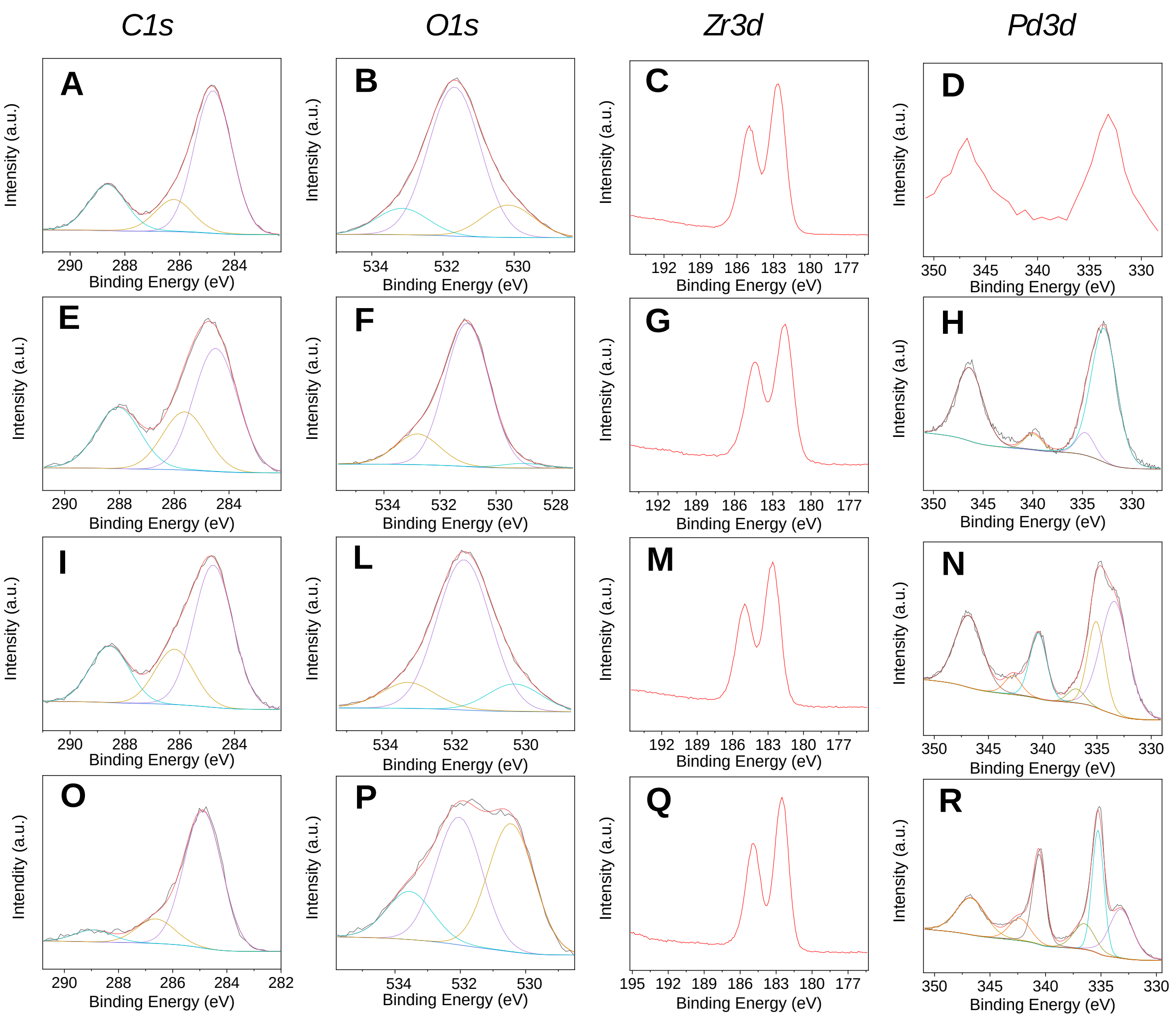

Figure S12: XPS spectra of **MOF-801** (A-D), **5Pd@MOF-801-1-200** (E-H), **5Pd@MOF-801 -2-200** (I-N), **5Pd@MOF-801 -2-500** (O-R). For each catalytic system, it is represented in this order the C1*s* (A, E, I, O), O1*s* (B, F, L, P), Zr3*d* (C, G, M, Q) and Pd3*d* (D, H, N, R) XPS regions.

CATALYTIC ACTIVITY

Hammet plot analysis

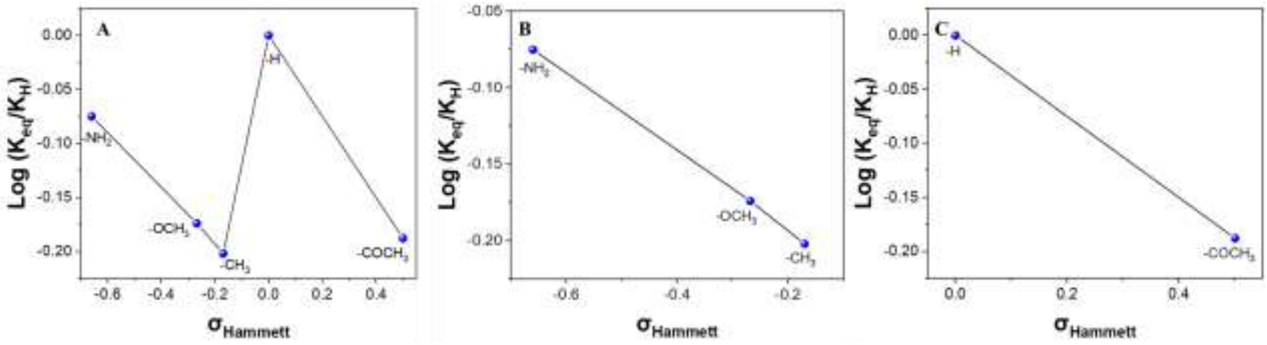

Figure S13. Hammett plot analysis of the Suzuki-Miyaura cross-coupling reaction catalyzed by 5Pd@UiO-66-NH_2-2-200.

TOF calculation

This section reports the full derivation of the TOF values used in the main text, including the calculation of the total number of Pd atoms in the catalyst, the average number of atoms per nanoparticle, the number of surface Pd atoms, and the site-normalized TON and TOF. All calculations are based on the optimized reaction conditions and the experimental TEM particle size (4.0 nm).

Total Amount of Pd in the Reaction

The optimized reaction used 2.5 mg of catalyst containing 5 wt% Pd.

$$m_{\mathrm{Pd}} = 2.5\ \mathrm{mg} \times 0.05 = 0.125\ \mathrm{mg} = 1.25 \times 10^{-4}\ \mathrm{g}$$

$$n_{\mathrm{Pd}} = \frac{m_{\mathrm{Pd}}}{M_{\mathrm{Pd}}} = \frac{1.25 \times 10^{-4}}{106.42} = 1.1746 \times 10^{-6}\ \mathrm{mol}$$

The corresponding number of Pd atoms is:

$$N_{\mathrm{Pd,total}} = n_{\mathrm{Pd}} \times N_A = (1.1746 \times 10^{-6})(6.022 \times 10^{23}) = 7.07 \times 10^{17}\ \mathrm{atoms}$$

Number of Pd Atoms per Nanoparticle

TEM analysis showed nanoparticles with an average diameter of 4.0 nm, radius:

$$r = 2.0 \times 10^{-9}\ \mathrm{m}$$

Nanoparticle volume

$$V_p = \frac{4}{3}\pi r^3 = \frac{4}{3}\pi(2.0 \times 10^{-9})^3 = 3.35 \times 10^{-26}\ \mathrm{m}^3$$

Atomic volume of Pd

$$V_{\mathrm{atom}} = \frac{M_{\mathrm{Pd}}}{\rho_{\mathrm{Pd}} N_A}$$

Using $\rho_{\mathrm{Pd}} = 12.02\ \mathrm{g\ cm^{-3}} = 1.202 \times 10^4\ \mathrm{kg\ m^{-3}}$:

$$V_{\mathrm{atom}} = \frac{106.42/1000}{(1.202 \times 10^4)(6.022 \times 10^{23})} = 1.47 \times 10^{-29}\ \mathrm{m}^3$$

Atoms per nanoparticle

$$N_{\mathrm{atoms/NP}} = \frac{V_p}{V_{\mathrm{atom}}} = \frac{3.35 \times 10^{-26}}{1.47 \times 10^{-29}} = 2.28 \times 10^3\ \mathrm{atoms\ per\ NP}$$

Total Number of Nanoparticles

$$N_{\mathrm{NP}} = \frac{N_{\mathrm{Pd,total}}}{N_{\mathrm{atoms/NP}}} = \frac{7.07 \times 10^{17}}{2.28 \times 10^3} = 3.09 \times 10^{14}\ \mathrm{nanoparticles}$$

Number of Surface Atoms per Nanoparticle

Surface area of one nanoparticle

$$A_p = 4\pi r^2 = 4\pi(2.0 \times 10^{-9})^2 = 5.03 \times 10^{-17}\ \mathrm{m}^2$$

Area occupied by one atom on Pd (111)

$$A_{\mathrm{atom,(111)}} = 1.31 \times 10^{-19}\ \mathrm{m}^2$$

Surface atoms per nanoparticle

$$N_{\mathrm{surf/NP}} = \frac{A_p}{A_{\mathrm{atom,(111)}}} = \frac{5.03 \times 10^{-17}}{1.31 \times 10^{-19}} = 3.84 \times 10^2\ \text{atoms}$$

Fraction of surface atoms

$$f_{\mathrm{surf}} = \frac{N_{\mathrm{surf/NP}}}{N_{\mathrm{atoms/NP}}} = \frac{384}{2280} = 0.17$$

Thus, 17% of all Pd atoms lie on the NP surface.

Total Moles of Surface Pd in the Reaction

$$N_{\mathrm{surf,total}} = N_{\mathrm{NP}} \times N_{\mathrm{surf/NP}} = (3.09 \times 10^{14})(3.84 \times 10^2) = 1.19 \times 10^{17}\ \text{surface atoms}$$
$$n_{\mathrm{surf}} = \frac{N_{\mathrm{surf,total}}}{N_A} = \frac{1.19 \times 10^{17}}{6.022 \times 10^{23}} = 1.97 \times 10^{-7}\ \mathrm{mol}$$

Product Formed and TON/TOF Values

The limiting reagent was iodobenzene (0.25 mmol).
A 93% yield gives:

$$n_{\mathrm{prod}} = 0.25 \times 10^{-3} \times 0.93 = 2.325 \times 10^{-4}\ \mathrm{mol}$$

Bulk-normalized TON

$$\mathrm{TON_{bulk}} = \frac{n_{\mathrm{prod}}}{n_{\mathrm{Pd}}} = \frac{2.325 \times 10^{-4}}{1.1746 \times 10^{-6}} = 197.9$$

Bulk TOF (reaction time = 40 min = 0.667 h)

$$\mathrm{TOF_{bulk}} = \frac{\mathrm{TON_{bulk}}}{0.667} = 2.97 \times 10^2\ \mathrm{h}^{-1}$$

Surface-Normalized TON and TOF

$$\mathrm{TON_{surf}} = \frac{n_{\mathrm{prod}}}{n_{\mathrm{surf}}} = \frac{2.325 \times 10^{-4}}{1.97 \times 10^{-7}} = 1.18 \times 10^3$$
$$\mathrm{TOF_{surf}} = \frac{\mathrm{TON_{surf}}}{0.667} = 1.76 \times 10^3\ \mathrm{h}^{-1}$$

TOF Assuming only 10% of surface Pd is catalytically active

If only edge/defect sites contribute to catalysis (10% of surface atoms):

$$n_{\text{active}} = 0.1\, n_{\text{surf}} = 1.97 \times 10^{-8}\ \text{mol}$$

$$\text{TON}_{\text{active}} = \frac{2.325 \times 10^{-4}}{1.97 \times 10^{-8}} = 1.18 \times 10^{4}$$

$$\text{TOF}_{\text{active}} = \frac{1.18 \times 10^{4}}{0.667} = 1.77 \times 10^{4}\ \text{h}^{-1}$$

Thus, the intrinsic TOF lies in the 10^4-10^5 h^{-1} range depending on the fraction of active surface atoms.

CHEM21 Metrics Calculations

The sustainability of the Suzuki-Miyaura cross-coupling catalysed by 5Pd@UiO-66-NH_2-2-200 was quantitatively assessed using the CHEM21 First Pass green metrics toolkit, which evaluates discovery-scale transformations through a combination of mass efficiency, energy input, solvent and reagent hazards, and work-up intensity. Key metrics were calculated following the standard definitions provided by the toolkit.

Atom Economy (AE)

AE is an intrinsic property of the transformation, independent of experimental yield, and is defined as:

$$\text{AE}\ (\%) = \frac{\text{MW}_{\text{product}}}{\sum \text{MW}_{\text{stoichiometric reagents}}} \times 100$$

MW product (biphenyl) = 154.21 g/mol

MW iodobenzene = 204.00 g/mol

MW phenylboronic acid = 121.93 g/mol

$$\text{AE} = \frac{154.21}{204.00 + 121.93} \times 100 = 47\%$$

Reaction Mass Efficiency (RME)

RME accounts for the actual experimental yield and is calculated as:

$$\text{RME}\ (\%) = \text{AE}\ (\%) \times \text{Yield}\ (\%)/100$$

- Experimental yield = 93%

$$\text{RME} = 47 \times \frac{93}{100} = 44\%$$

Overall Efficiency (OE)

OE quantifies the fraction of the mass of reagents converted to product relative to the theoretical efficiency:

$$\text{OE}\ (\%) = \frac{\text{RME}}{\text{AE}} \times 100$$

$$\text{OE} = \frac{44}{47} \times 100 = 93.6\%$$

Process Mass Intensity (PMI)

$$\text{PMI} = \frac{\text{total mass of input materials}}{\text{mass of isolated product}}$$

- Iodobenzene: 0.25 mmol × 204.00 g/mol = 0.0510 g
- Phenylboronic acid: 0.30 mmol × 121.93 g/mol = 0.0366 g
- K_2CO_3: 0.50 mmol × 138.21 g/mol = 0.0691 g
- Catalyst: 2.5 mg = 0.0025 g
- EtOH (3 mL, ρ = 0.789 g/mL) = 2.367 g

Total mass in = 2.5262 g
Mass of product = 0.233 mmol x 154.21 g/mol = 0.0359 g

$$\text{PMI} = \frac{2.5262}{0.0359} = 70$$

Solvent Contribution to PMI

$$\text{Solvent fraction } = \frac{\text{mass of EtOH}}{\text{total mass input}} = \frac{2.367}{2.5262} = 0.937$$

Thus, almost 94% of the mass input originates from solvent, consistent with typical small-scale discovery reactions.

Summary of Metrics for Radar Chart

Metric	Value
Conversion	100%
Yield	93%
Selectivity	100%
OE	93.6%
RME	44%
AE	47%

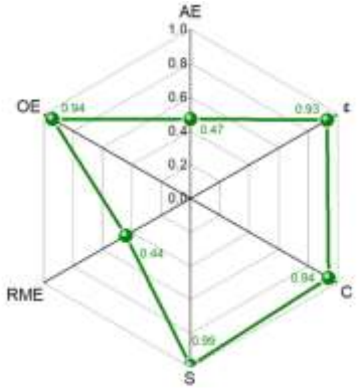

Notes for visualization: All values can be normalized 0-1 for radar chart representation. Conversion, yield, selectivity, and OE are effectively green-flag; RME and AE reflect the influence of solvent on mass efficiency.

Electrocatalytic HER

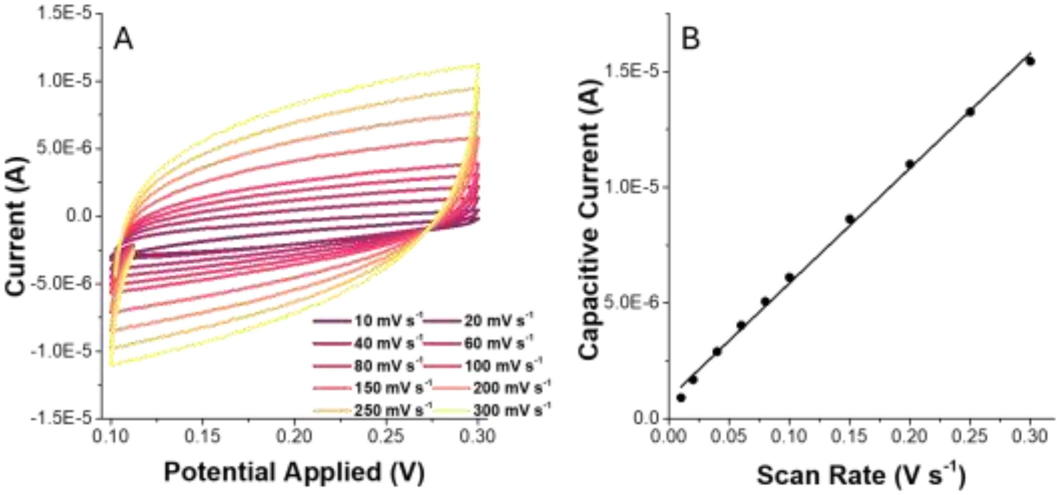

Figure S14. A) Cyclic voltammograms at different scan rates recorded with a GCE modified with 5Pd@C; B) Linear fit of the capacitive current for the determination of ECSA. The voltammograms were recorded in H_2SO_4 0.5 M, using an Ag/AgCl 3M reference electrode and a Pt wire as counter electrode.

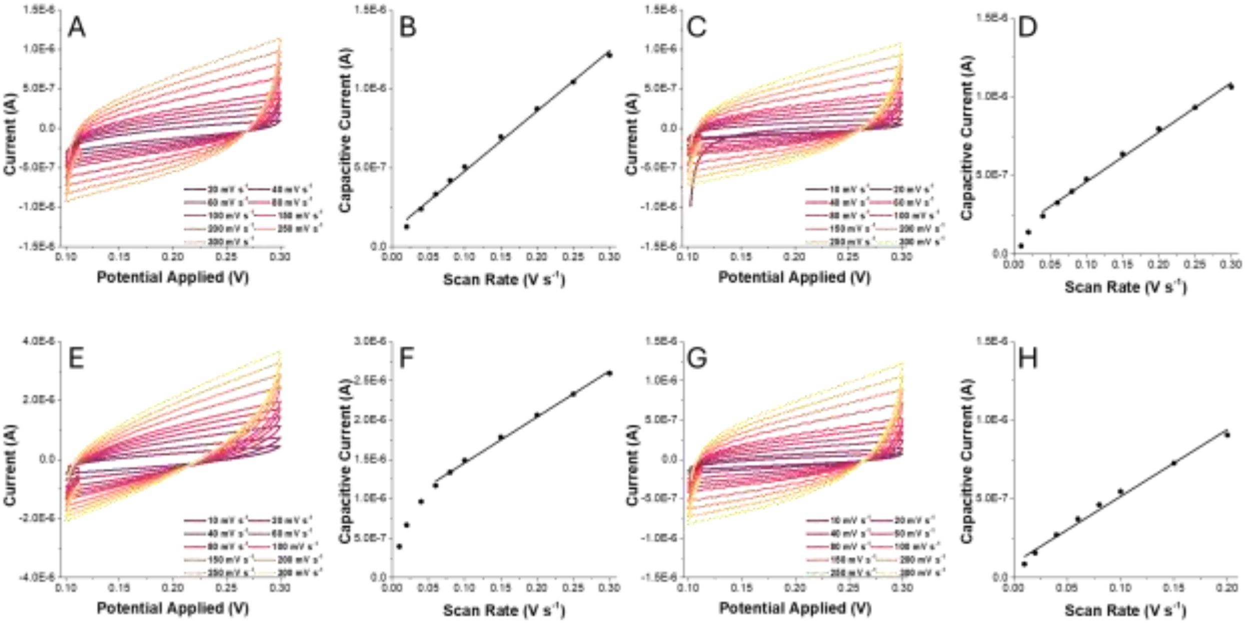

Figure S15. Cyclic voltammograms at different scan rates and linear fit of the capacitive currents for the determination of ECSA of GCE modified with 5Pd@MOF-801-1-200 (A-B), 5Pd@MOF-801-1-500 (C-D), 5Pd@MOF-801-2-200 (E-F), and 5Pd@MOF-801-2-500 (G-H). The voltammograms were recorded in H_2SO_4 0.5 M, using an Ag/AgCl 3M reference electrode and a Pt wire as counter electrode.

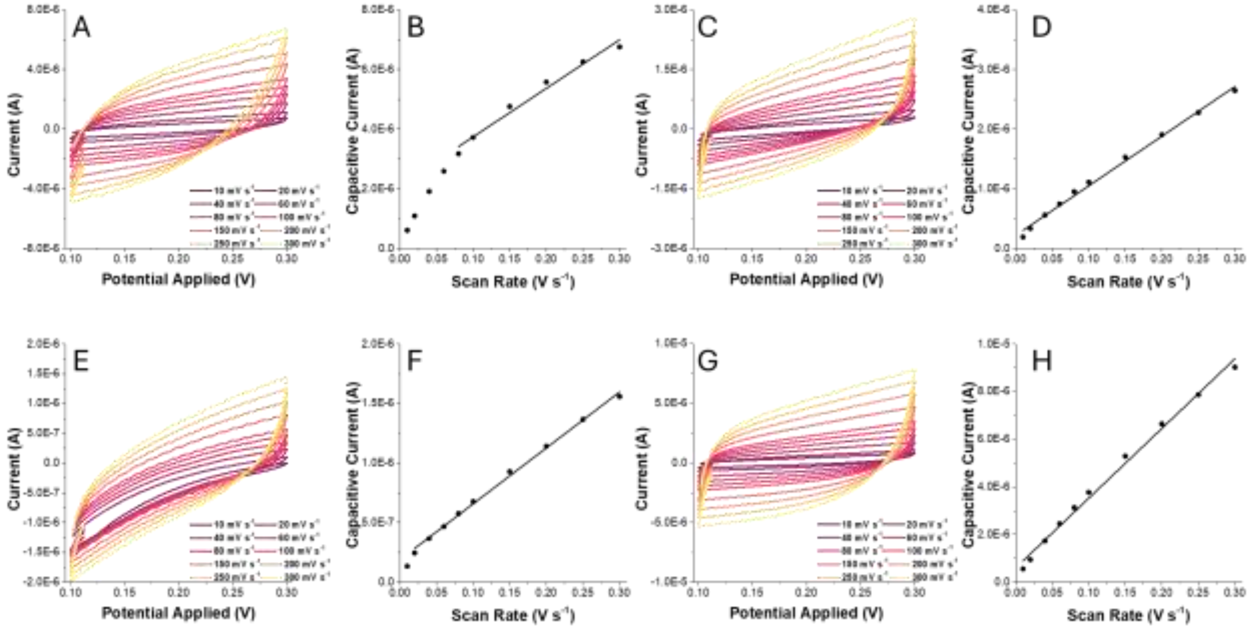

Figure S16. Cyclic voltammograms at different scan rates and linear fit of the capacitive currents for the determination of ECSA of GCE modified with 5Pd@UiO66-NH_2-1-200 (A-B), 5Pd@ UiO66-NH_2-1-500 (C-D), 5Pd@ UiO66-NH_2-2-200 (E-F), and 5Pd@ UiO66-NH_2-2-500 (G-H). The voltammograms were recorded in H_2SO_4 0.5 M, using an Ag/AgCl 3M reference electrode and a Pt wire as counter electrode.

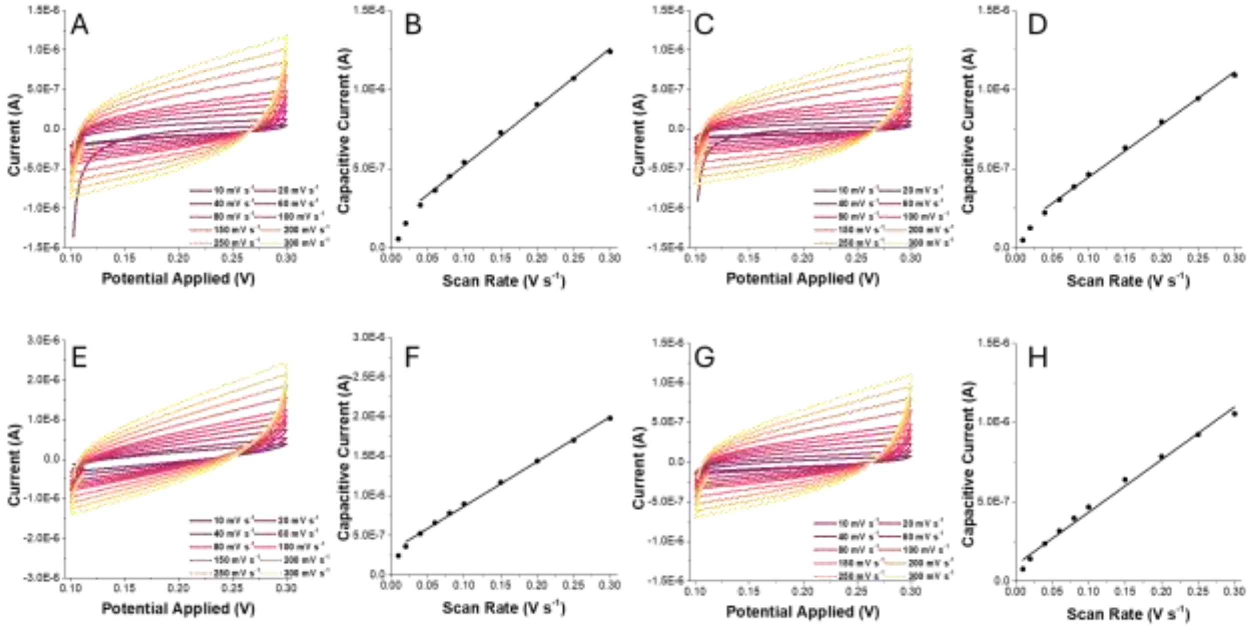

Figure S17. Cyclic voltammograms at different scan rates and linear fit of the capacitive currents for the determination of ECSA of GCE modified with 5Pd@UiO66-1-200 (A-B), 5Pd@ UiO66-1-500 (C-D), 5Pd@ UiO66-2-200 (E-F), and 5Pd@ UiO66-2-500 (G-H). The voltammograms were recorded in H_2SO_4 0.5 M, using an Ag/AgCl 3M reference electrode and a Pt wire as counter electrode.

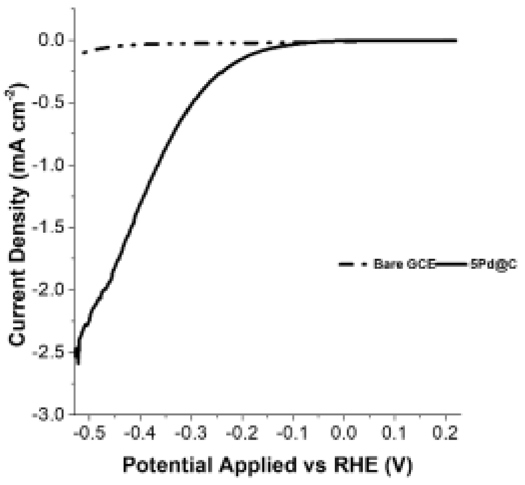

Figure S18. Voltammograms recorded with the bare GCE electrode as negative control (dotted line) and with a GCE modified with 5Pd@C as positive control (continuous line). The voltammograms were recorded in H_2SO_4 0.5 M, using an Ag/AgCl 3M reference electrode and a Pt wire as counter electrode. The potential was swept with a scan rate of 2 mV s^{-1} from 0 to -0.75 V vs Ag/AgCl. The potential was then corrected to the RHE scale.

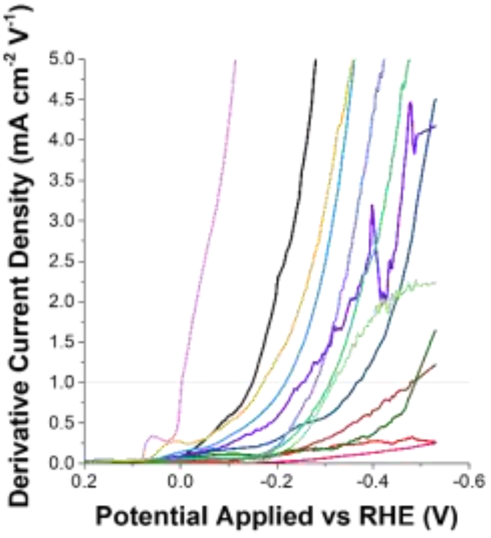

Figure S19. First-order derivative of the current density as a function of the applied potential. The LSV were first smoothed with a Savitzky-Golay method, using a 35-pts window size and a 2nd grade polynomial fit. The onset potential was defined as the potential at which $dj/dV \geq 1$mA cm^{-2} V^{-1}.

Table S2: Values of Onset Potential, Tafel Slope, Tafel Constant, and Exchange Current Density for all the modified electrodes.

	Onset Potential	Current Density at -0.5 V $\|j\|_{\eta=-0.5}$	Tafel Slope	Tafel Constant	Exchange Current Density
	V	mA cm^{-2}	mV dec^{-1}	V	µA cm^{-2}
**5@PdC**	-0.148	2.24	-160	-0.333	8.27
**5Pd@MOF801-1-200**	-0.220	2.13	-240	-0.672	1.60
**5Pd@MOF801-1-500**	-0.366	0.39	-238	-0.719	0.94
**5Pd@MOF801-2-200**	-0.281	0.95	-178	-0.479	2.08
**5Pd@MOF801-2-500**	-0.253	0.67	-248	-0.496	9.98
**5Pd@UiO66-NH$_2$-1-200**	-	0.08	-311	-0.783	3.03
**5Pd@UiO66-NH$_2$-1-500**	-0.478	0.17	-287	-0.709	3.39
**5Pd@UiO66-NH$_2$-2-200**	-0.002	13.94	-158	-0.187	65.54
**5Pd@UiO66-NH$_2$-2-500**	-	0.03	-297	-0.935	0.73
**5Pd@UiO66-1-200**	-0.317	0.38	-592	-1.260	7.76
**5Pd@UiO66-1-500**	-0.488	0.13	-457	-0.929	9.31
**5Pd@UiO66-2-200**	-0.308	0.66	-191	-0.517	1.95
**5Pd@UiO66-2-500**	-0.170	2.00	-250	-0.417	21.43

NMR AND MASS SPECTRA

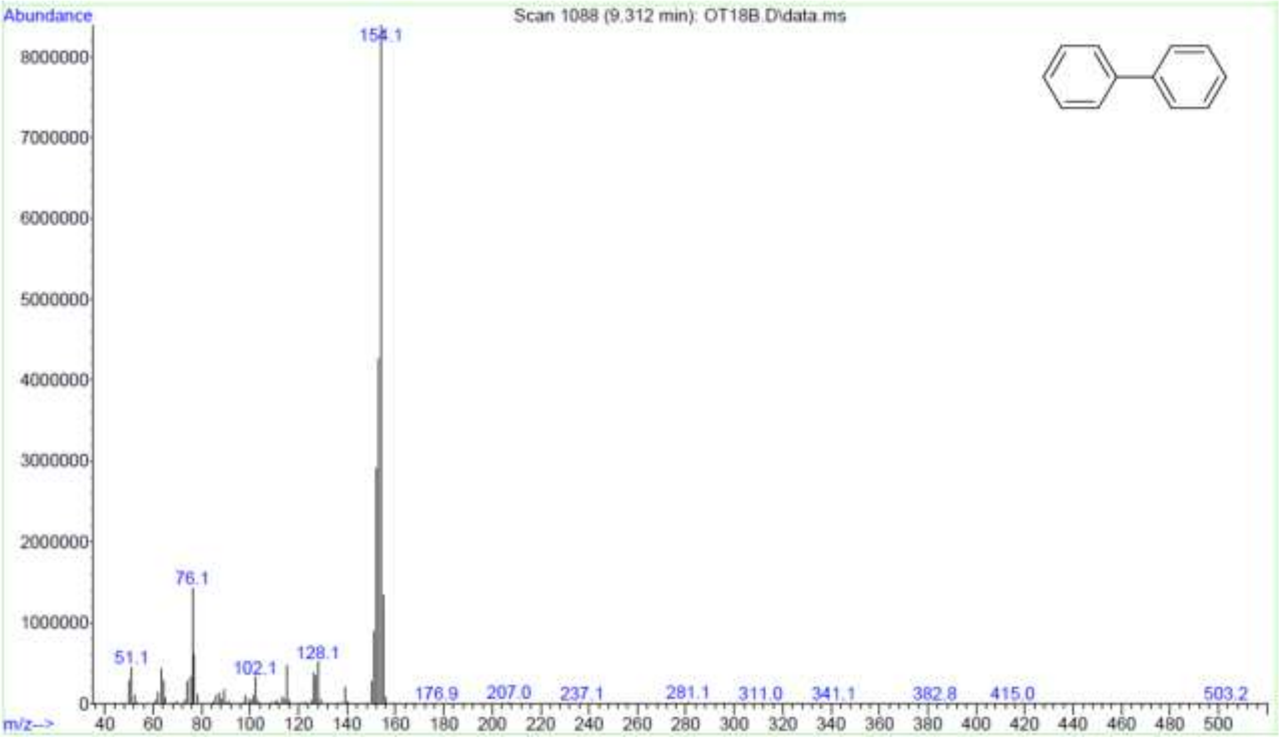

Figure A6. *Mass spectrum (EI, 70eV) of biphenyl.*

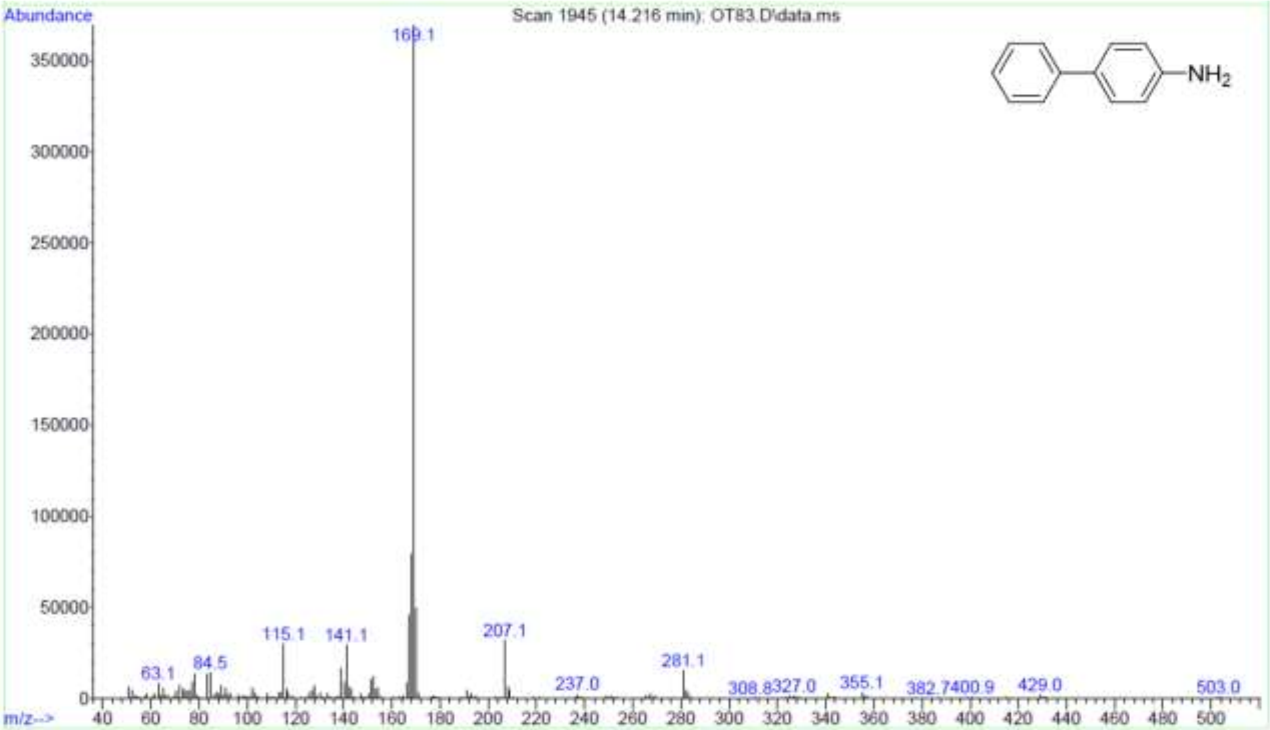

Figure A7. *Mass spectrum (EI, 70eV) of 4-aminobiphenyl.*

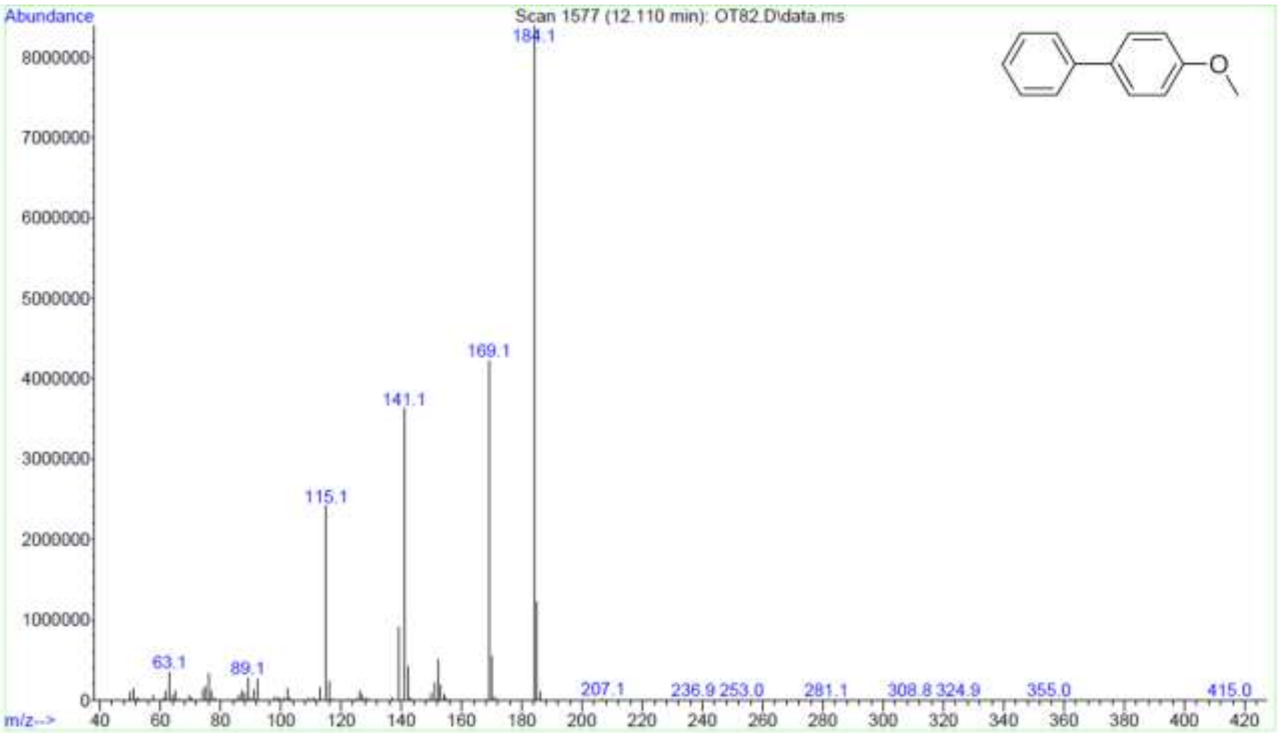

Figure A8: *Mass spectrum (EI, 70eV) of 4-Methoxybiphenyl.*

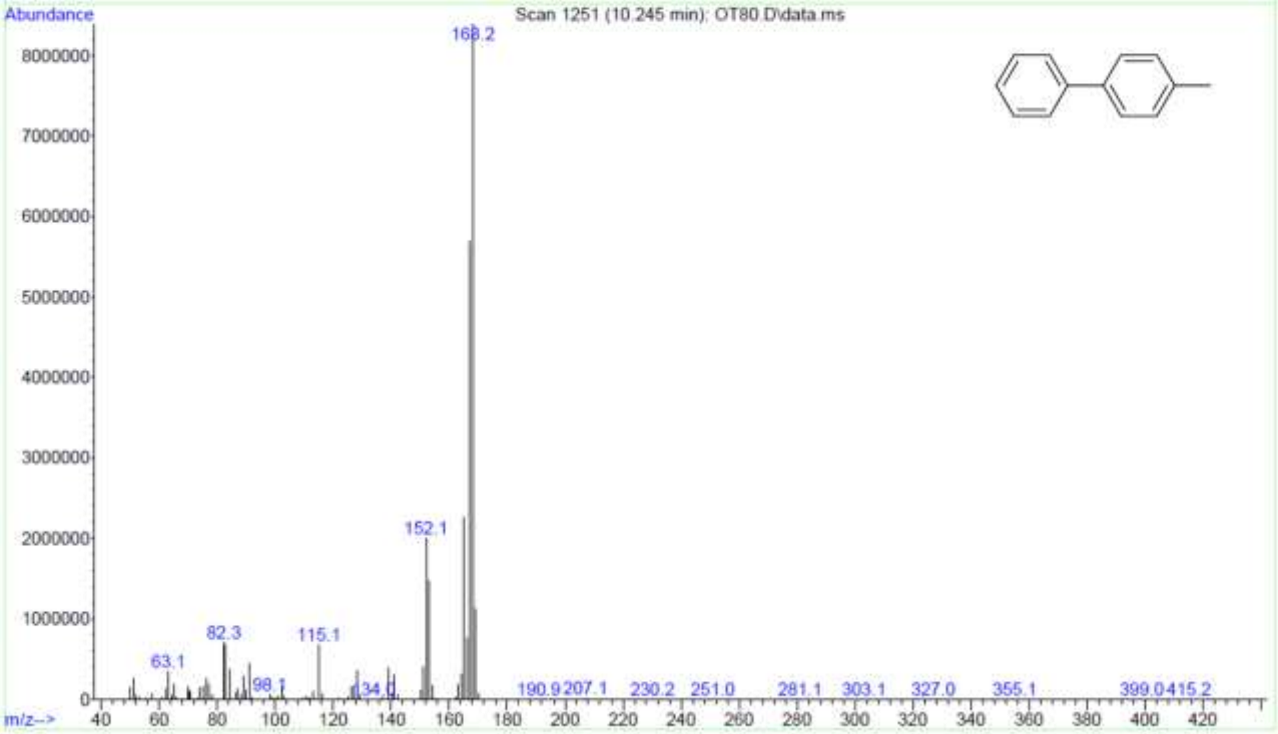

Figure A9: *Mass spectrum (EI, 70eV) of 4-Methylbiphenyl.*

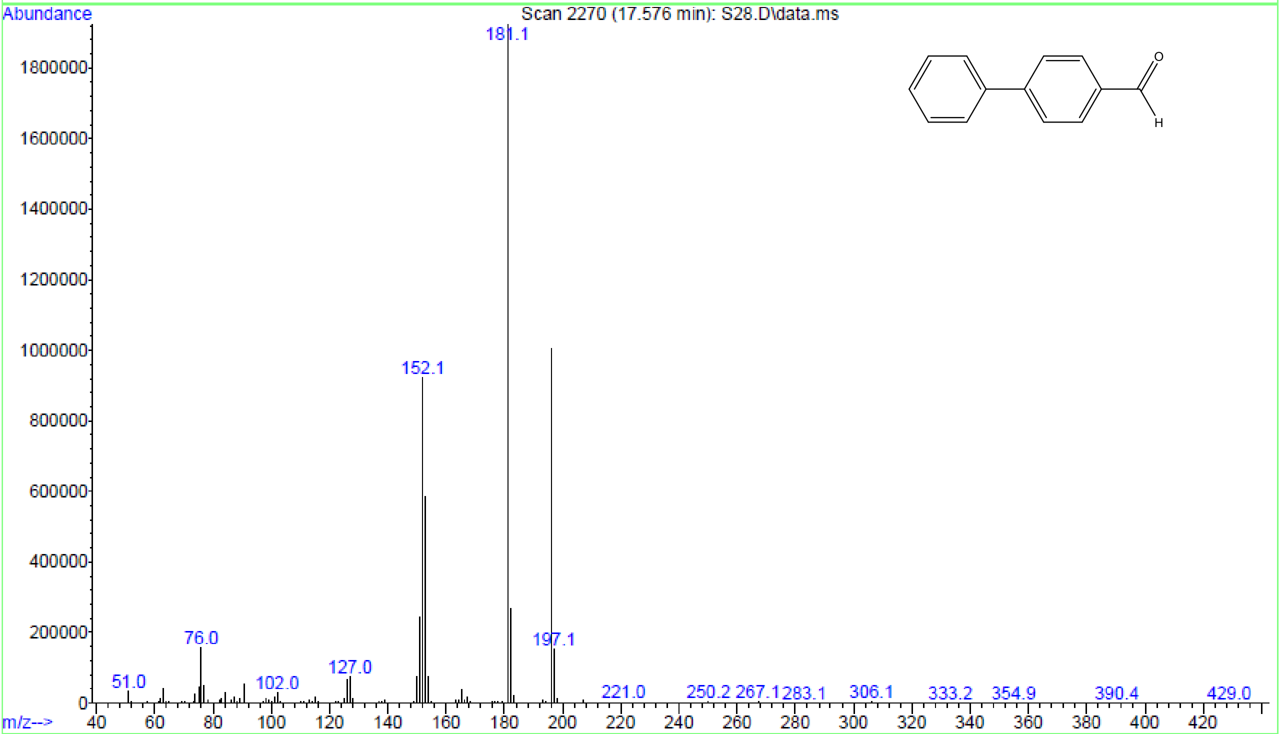

Figure A10. *Mass spectrum (EI, 70eV) of 4-Phenylbenzaldehyde.*

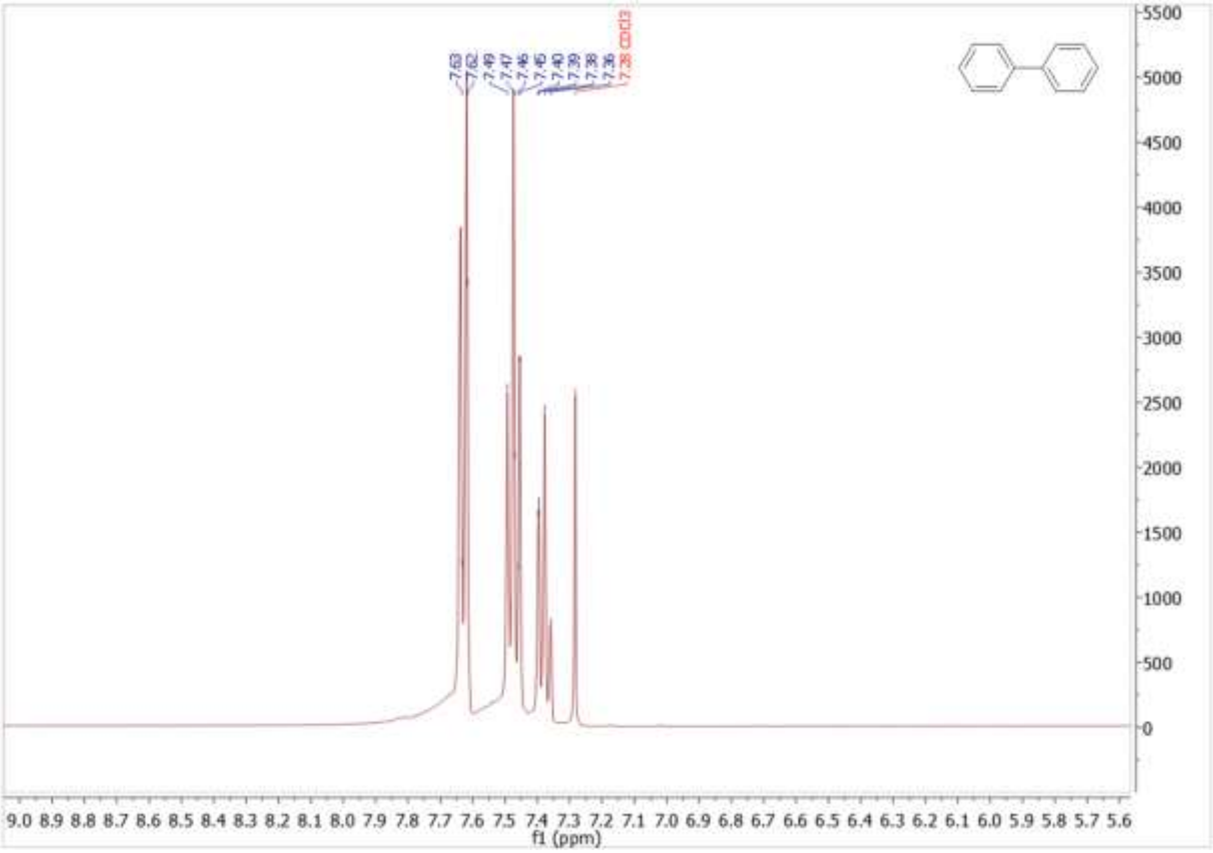

Figure A13. *^{1}H NMR (400 MHz, $CDCl_3$, 298K) of Biphenyl.*

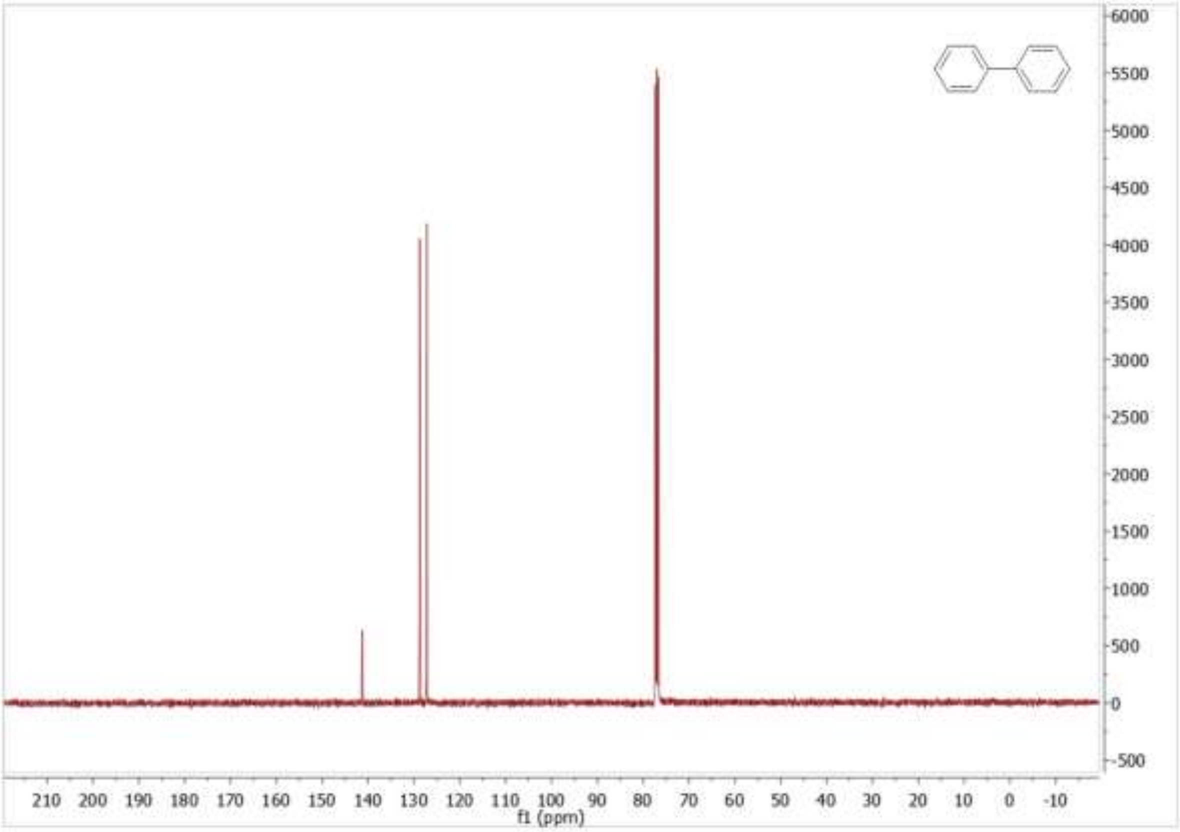

***Figure A14**: ^{13}C NMR (100 MHz, $CDCl_3$, 298K) δ 141.25, 128.75, 127.25. Compound Biphenyl.*
